# How the venetian blind percept emerges from the laminar cortical dynamics of 3D vision

**DOI:** 10.3389/fpsyg.2014.00694

**Published:** 2014-08-05

**Authors:** Yongqiang Cao, Stephen Grossberg

**Affiliations:** ^1^HRL Laboratories, LLCMalibu, CA, USA; ^2^Graduate Program in Cognitive and Neural Systems, Department of Mathematics, Center for Adaptive Systems, Center for Computational Neuroscience and Neural Technology, Boston UniversityBoston, MA, USA

**Keywords:** Venetian blind effect, visual cortex, stereopsis, binocular vision, perceptual grouping, surface perception, consciousness, LAMINART model

## Abstract

The 3D LAMINART model of 3D vision and figure-ground perception is used to explain and simulate a key example of the Venetian blind effect and to show how it is related to other well-known perceptual phenomena such as Panum's limiting case. The model proposes how lateral geniculate nucleus (LGN) and hierarchically organized laminar circuits in cortical areas V1, V2, and V4 interact to control processes of 3D boundary formation and surface filling-in that simulate many properties of 3D vision percepts, notably consciously seen surface percepts, which are predicted to arise when filled-in surface representations are integrated into surface-shroud resonances between visual and parietal cortex. Interactions between layers 4, 3B, and 2/3 in V1 and V2 carry out stereopsis and 3D boundary formation. Both binocular and monocular information combine to form 3D boundary and surface representations. Surface contour surface-to-boundary feedback from V2 thin stripes to V2 pale stripes combines computationally complementary boundary and surface formation properties, leading to a single consistent percept, while also eliminating redundant 3D boundaries, and triggering figure-ground perception. False binocular boundary matches are eliminated by Gestalt grouping properties during boundary formation. In particular, a disparity filter, which helps to solve the Correspondence Problem by eliminating false matches, is predicted to be realized as part of the boundary grouping process in layer 2/3 of cortical area V2. The model has been used to simulate the consciously seen 3D surface percepts in 18 psychophysical experiments. These percepts include the Venetian blind effect, Panum's limiting case, contrast variations of dichoptic masking and the correspondence problem, the effect of interocular contrast differences on stereoacuity, stereopsis with polarity-reversed stereograms, da Vinci stereopsis, and perceptual closure. These model mechanisms have also simulated properties of 3D neon color spreading, binocular rivalry, 3D Necker cube, and many examples of 3D figure-ground separation.

## 1. Introduction: design principles for how the brain sees the world in depth

### 1.1. Explaining 3D percepts using laminar cortical networks

**Figure 1:**
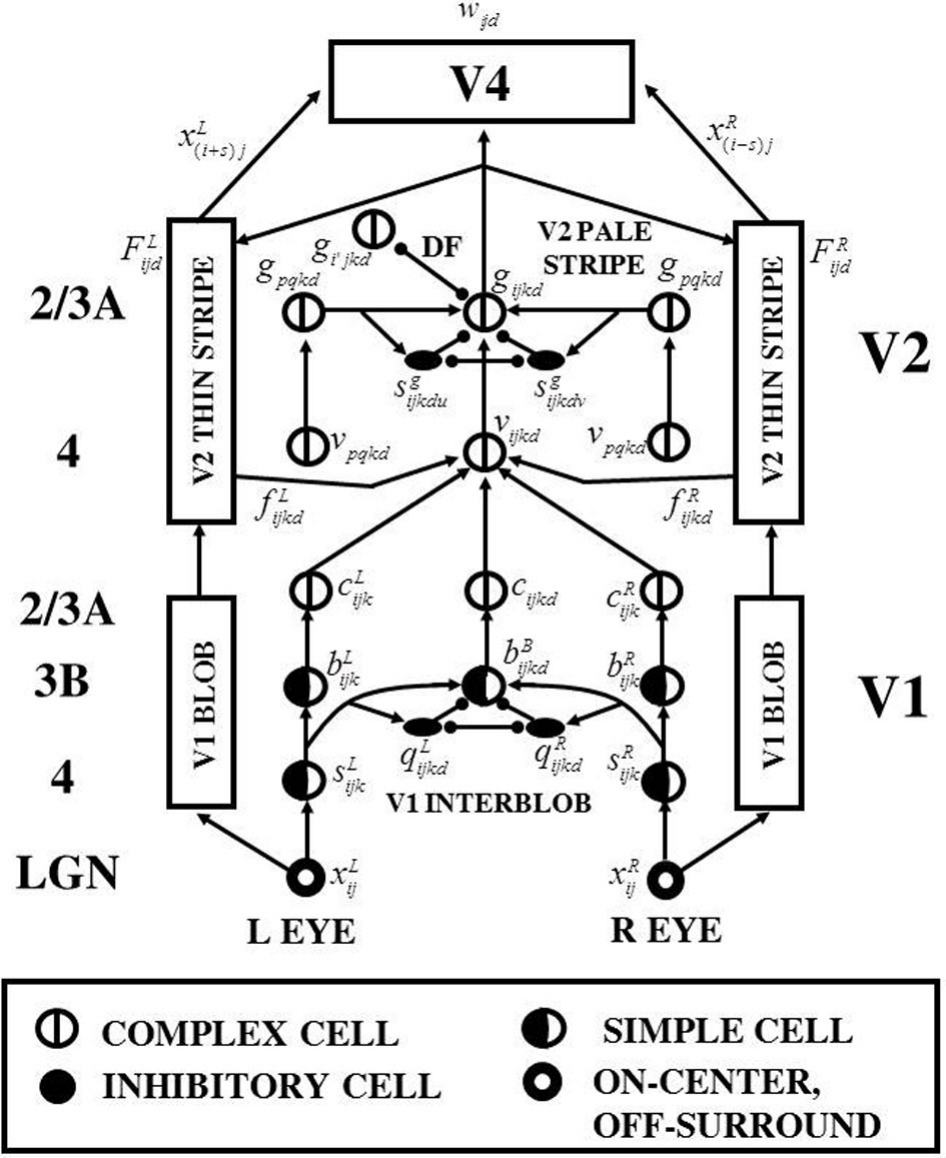
**3D LAMINART model variables, circuits, and processing stages**. The model consists of interactions between a boundary cortical stream and a surface cortical stream. The boundary stream computes 3D perceptual groupings. Its processing stages are in the middle of the figure, and go through the cortical areas (V1 Interblob)—(V2 Pale Stripe)—V4. The surface stream computes 3D surface representations of lightness, color, and depth. Its processing stages flank those of the boundary stream on both the left and right, and go through the cortical areas (V1 Blob)—(V2 Thin Stripe)—V4. The cortical layers in which the various processes are hypothesized to take place are listed in the left column of the figure. The mathematical variables of the various model processing stages provide a visualization of the network dynamics that are mathematically defined in Section 4. Adapted with permission from Cao and Grossberg (2005).

The 3D LAMINART model (Figure 1) predicts how the LGN and cortical areas V1, V2, and V4 computed monocular and binocular visual information to produce three-dimensional (3D) boundary groupings and conscious surface percepts. Each cell type in the model clarifies anatomical and neurophysiological data. Grossberg and Howe (2003), Grossberg and Yazdanbakhsh (2005), and Raizada and Grossberg (2003) provide reviews.

Among the percepts that the model can simulate is the Venetian blind percept that was described in Figure 6.21 of Howard and Rogers (1995). This Venetian blind stimulus consists of two gratings. A low frequency grating is presented to the left eye, whereas a high frequency one presented to the right eye. During binocular fusion, every second bar of the left grating is in retinal correspondence with every third bar of the right grating. The resulting percept consists of short ramps. Each ramp contains three bars that slope up from left to right. These ramps are interspaced with step returns.

Cao and Grossberg (2005, 2012) showed how the 3D LAMINART model could quantitatively simulate the surface properties that are consciously seen in 18 challenging psychophysical experiments, a feat still not matched by competing models, and did so with a single set of model equations and parameters (see Section 4). The implementation of the model with spiking neurons in Cao and Grossberg (2012) also simulated these surface percepts and demonstrated how analog properties of these percepts emerge from the interactive dynamics of discrete spikes. A simulation of the Venetian blind percept was one of the 18 simulated experiences in these articles, but was only briefly explained. The percept was also simulated using an earlier version of the model by Grossberg and Howe (2003). The current article provides a detailed, step-by-step explanation of the percept and shows that its explanation uses the same combination of laminar cortical mechanisms that can be used to explain percepts like Panum's limiting case (Panum, 1858; Gillam et al., 1995; McKee et al., 1995), where a bar presented to one eye is simultaneously matched to two separate bars presented to the other eye. Also see Grossberg and Howe (2003).

In order to provide a self-contained exposition, much of the text is revised and refined from Cao and Grossberg (2005, 2012), with additional text and figures added to explain the Venetian blind effect in greater detail. This section heuristically describes the seven organizational principles governing the model's design. Section 2 provides a functional model description without mathematical equations. Section 3 summarizes simulations of the Venetian blind effect and Panum's limiting case, and clarifies how they are connected. Section 4 provides a mathematical description of model equations and parameters. Sections 2 and 4 are written to enable the functional and mathematical model descriptions to be coordinated with each other and the model diagrams. Section 5 provides a discussion of how the Venetian blind and Panum's limiting case simulations fit into a larger theory of the cortical dynamics that are predicted to support attention, search, learning, recognition, and conscious awareness of these visual percepts. The reader can skip from Section 3 to 5 for this discussion, should the mathematical equations not be of primary concern.

### 1.2. How the brain sees in depth: seven brain designs

**Figure 2:**
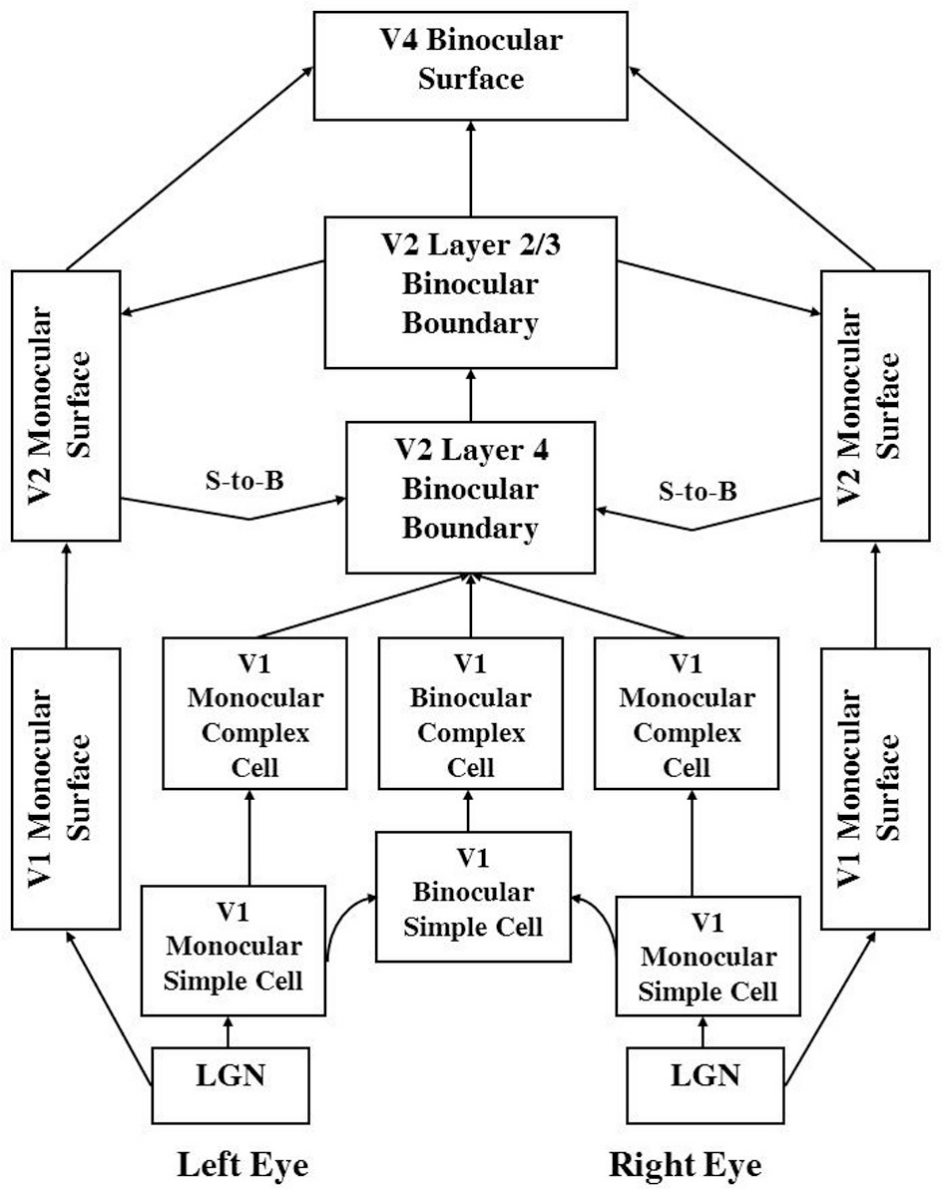
**3D LAMINART functional macrocircuit**. This figure provides functional names of the processes that are shown in Figure 1. In order to clarify which functional name corresponds to which mathematical variables and anatomical processing stages in Figure 1, the connections in this figure are drawn to mimmick those in Figure 1. The abbreviation S-to-B stands for Surface-to-Boundary feedback signals. These functional names are used to define the mathematical variables in Section 4. They are also used to label the processing stages in the simulation **Figures 6–10**. Taken together, Figure 1, and this figure and Section 4 should enable the reader to track each variable that was simulated, as well as the mathematical equation that determines its dynamics through time.

The 3D LAMINART model achieves its explanatory power by embodying seven functional designs into its laminar cortical circuitry. Figures 1 and 2 depict this circuitry, whose operation is intuitively explained in Section 2.

#### 1.2.1. How contrast-specific binocular fusion coexists with contrast-invariant boundary perception

**Figure 3:**
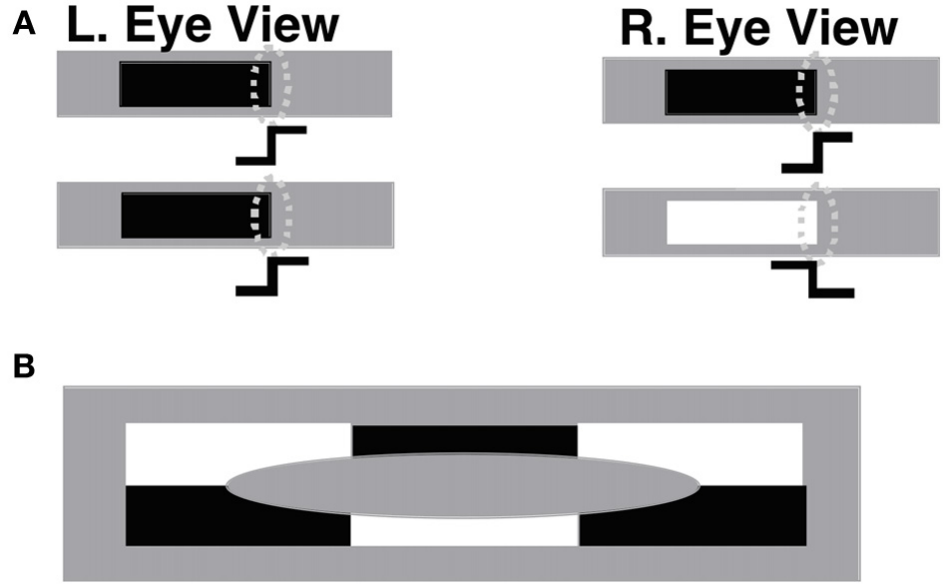
**(A)** The same-sign hypothesis: only edges that have the same contrast polarity can be stereoscopically fused to produce a percept of depth. The rightmost edges in the left and right eye figures in the upper row, which have the same contrast polarity and orientation, can be binocularly fused if their corresponding simple cells (oval shapes) input to complex cells with an appropriate disparity sensitivity. The rightmost edges in the left and right eye figures in the lower row, having opposite contrast polarities, cannot be binocularly fused. **(B)** Individual complex cells receive inputs from simple cells that respond to both dark-light and light-dark contrast polarities, as well as to both red-green and green-red, and blue-yellow and yellow-blue contrasts. As a result, complex cells can respond in a contrast-invariant way along the boundary of a figure whose contrast polarity reverses as the boundary is traversed. In particular, complex cells can respond at every position along the boundary of the depicted ellipse, even though the contrast polarity reverses relative to the background as its perimeter is traversed. Reproduced with permission from Cao and Grossberg (2005).

Veridical stereoscopic depth perception depends on binocularly fusing only pairs of edge signals from the left and right retinal images that belong to the same object. This is commonly referred to as the *Correspondence Problem* (Julesz, 1971; Howard and Rogers, 1995). One step in solving this problem is to allow binocular fusion to occur only between edge signals from the left and right retinal images that have the same contrast polarity. Binocular fusion thus obeys the *same-sign hypothesis* (Figure 3A). In addition, fused boundaries form around objects whose contrast polarities on their bounding contours reverse along their perimeters (Figure 3B; Grossberg, 1994); that is, are contrast-invariant. Both constraints are realized in the model by interactions between orientationally tuned cells in layers 4, 3B, and 2/3A of cortical area V1 interblobs (Figure 1).

#### 1.2.2. Contrast magnitude constraint on binocular fusion

Another step in solving the Correspondence Problem is to binocularly fuse only edges with approximately the same magnitude of contrast (McKee et al., 1994). This constraint, called the *obligate property* (Poggio, 1991), emerges in the model through interactions between excitatory and inhibitory cells in layer 3B of V1 (Figure 1). A mathematical proof of the obligate property in the model is provided in Section 4.

#### 1.2.3. Disparity filter

**Figure 4:**
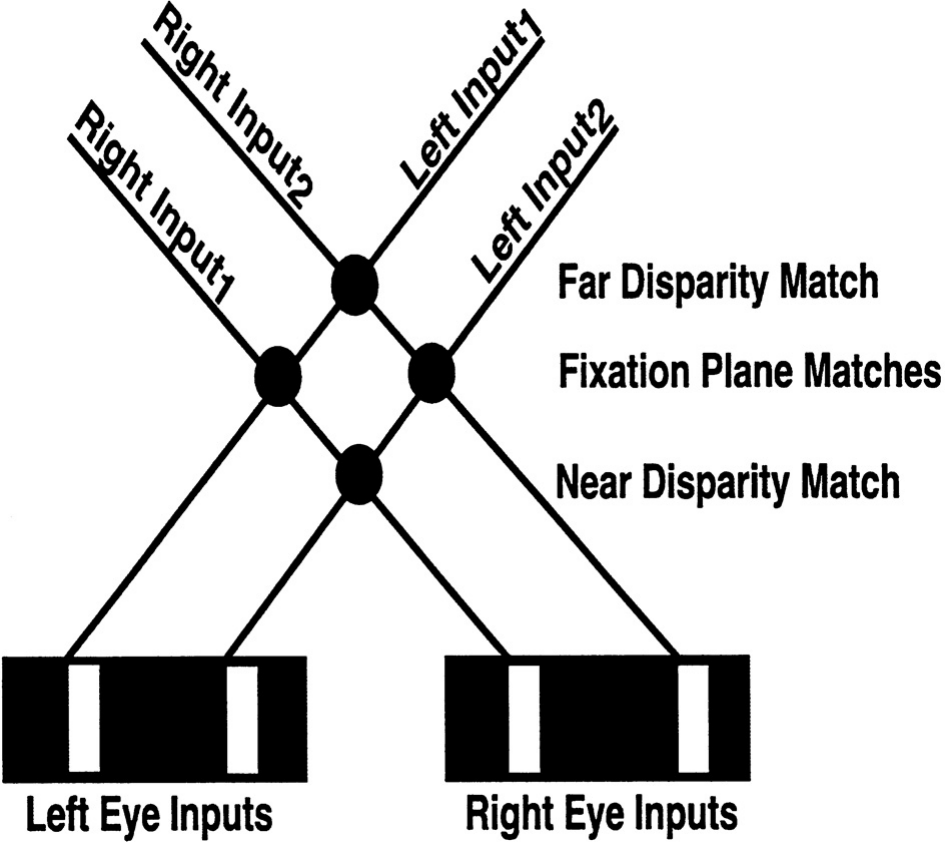
**Binocular matching and the Correspondence Problem**. The V1 binocular boundary network matches an edge in one retinal image with every other edge in the other retinal image whose relative disparity is not too great, that has the same contrast polarity, and whose magnitude of contrast is not too different. In response to an image seen in depth with two white vertical bars on a black background, the V1 boundary network creates four matches, with the two not in the fixation plane being false matches between edges that do not correspond to the same object. As described in the text, these false matches are suppressed by the disparity filter in V2, wherein each neuron is inhibited by every other neuron that shares either of its monocular inputs (i.e., shares a monocular line-of-sight represented by the solid lines; “line-of-sight inhibition”). Note in particular that the solid lines that represent the monocular lines-of-sight also enable the computation of allelotropic shifts (see **Table 1**): an edge in the left retinal image is shifted to the right for matches increasingly further away, whereas an edge in the right retinal image is shifted in the opposite direction. Reproduced with permission from Cao and Grossberg (2005).

Many false binocular matches can still occur that do not derive from the same objects (Figure 4). Some authors attempt to eliminate these false matches by imposing a *unique-matching rule*, whereby each feature in one retinal image is matched with at most one feature in the other retinal image (Marr and Poggio, 1976; Grimson, 1981). However, this rule fails to explain critical psychophysical data, such as the percept that arises in Panum's limiting case (Panum, 1858; Gillam et al., 1995; McKee et al., 1995). In this percept, a bar presented to one eye is simultaneously matched to two bars presented to the other eye. The unique-matching rule also fails in the Venetian Blind effect. The 3D LAMINART model does not impose unique matches. Rather, correct matches are facilitated by using a *disparity filter* (see DF in Figure 1; Grossberg and McLoughlin, 1997; McLoughlin and Grossberg, 1998), whose circuit uses line-of-sight inhibition between active cells that represent different depths at the same position. A parsimonious property of the model, and one that suggests how this constraint may have arisen during evolution, is that these inhibitory interactions are part of the perceptual grouping process that selects and completes 3D boundaries within cortical layer 2/3 (see Section 1.2.5).

#### 1.2.4. Monocular and binocular information combine in forming depth percepts

Panum's limiting case has homologs in many naturally occurring situations where one edge seen by one eye and two possible edges with which to match it are seen by the other eye. For example, an object's edge seen by one eye may be occluded by a nearer object when viewed by the other eye. This happens during da Vinci stereopsis (Nakayama and Shimojo, 1990; Gillam et al., 1999). The monocularly viewed region nonetheless has a definite depth that is induced by binocularly viewed scenic features. Monocular information can hereby contribute to forming seamless depthful percepts. These monocular-binocular interactions have been probed by varying the relative contrast of the bars in Panum's limiting case displays and thereby causing alterations in the ensuing percepts of depth (Smallman and McKee, 1995). Another type of display that is useful for the study of monocular-binocular interactions is dichoptic masking, where an object presented to one eye is masked by one presented to the other eye (McKee et al., 1994). All of these variations have been simulated using the 3D LAMINART model.

How do monocular boundaries contribute to depthful percepts? To which depths should a monocular boundary be assigned? This *Monocular-Binocular Interface Problem* was analyzed in Grossberg (1994, 1997) as part of the larger problem of explaining 3D figure-ground percepts. The proposed solution was first implemented in simulations by Grossberg and Howe (2003) of many data about 3D surface perception. This hypothesis predicts that the outputs of monocular boundary cells are added to all depth planes in cortical area V2 pale stripes along their respective lines-of-sight. Layer 4 is a likely site for combining monocular and binocular boundary signals from V1 (Figure 4). Yazdanbakhsh and Watanabe (2004) successfully tested this prediction by doing psychophysical experiments on human subjects. Monocular boundaries that are not at depths where they can form closed boundaries are eliminated by the disparity filter, which is predicted to occur in V2 pale stripes in layer 2/3, where binocular grouping is completed, as explained below. The Monocular-Binocular Interface Problem is hereby resolved at the disparity filter, even as it helps to solve the Correspondence Problem.

#### 1.2.5. 3D perceptual groupings eliminate false matches

Interactions between pyramidal cells in layer 2/3 of the V2 pale stripes were predicted to carry out perceptual grouping using a *bipole property*, This property utilizes both long-range excitatory recurrent axons and shorter-range inhibitory axons. The long-range excitatory recurrent axons connect cells that are (approximately) colinear and coaxial with respect to one another across space. For example, if a Kanizsa square image is presented, each pair of collinear pacmen edges can activate pyramidal cells with like-oriented long-range horizontal connections whose signals summate on target cells between the pacmen. These long-range horizontal connections also activate the shorter-range inhibitory interneurons. These interneurons inhibit each other and nearby pyramidal cells (Figure 1). Due to their mutual inhibition, the total activity in their local inhibitory network is normalized; cf. Grossberg (1973). When the total summating excitation from the long-range excitatory axons on both sides of a target cell, combines with the total normalized inhibition from the shorter-range inhibitory axons, the net excitation can fire the cells cf., von der Heydt and Peterhans (1989) and von der Heydt et al. (1984). This balance of “two-against-one” of excitation and inhibition at target cells implements the bipole property (Grossberg et al., 1997; Grossberg and Raizada, 2000; Raizada and Grossberg, 2001).

When only a single pacman is the inducing stimulus, it induces long-range excitation and disynaptic inhibition from only one side of a recipient cell. In this case, the inhibition is not normalized, so that the excitation and inhibition are approximately balanced, a case of “one-against-one” excitation vs. inhibition, so the target cell is not excited. If the cell receives bottom-up input, it can activate the cell without inputs from any long-range excitatory axons, but its activity can be modulated by such inputs (Bringuier et al., 1999; Crook et al., 2002). Excitatory modulations also help to guide the spread of attention along a boundary grouping (Roelfsema et al., 1998; Ito and Gilbert, 1999; Roelfsema and Spekreijse, 1999; Grossberg and Raizada, 2000), the grouping of 2D and 3D planar percepts (Kapadia et al., 1995; Polat et al., 1998; Bakin et al., 2000), and the grouping of 3D slanted and curved percepts (Grossberg and Swaminathan, 2004).

The 3D LAMINART model predicts that the shorter-range inhibitory interneurons also inhibit the pyramidal cells that correspond to other orientations, notably perpendicular orientations, thereby contributing to figure-ground and binocular rivalry percepts, among others (Grossberg and Swaminathan, 2004; Cao and Grossberg, 2005, 2012; Grossberg and Yazdanbakhsh, 2005; Grossberg et al., 2008). As noted in the previous section, some of these inhibitory interneurons also realize the disparity filter as part of the grouping process. The model hereby predicts that *the selection of a correct 3D grouping includes the suppression of false binocular matches*. The model hereby shows how the solution of the Correspondence Problem also helps to solve the Gestalt grouping problem.

During depth perception, the perceived depths of emergent perceptual groupings, such as illusory contours, often covary with the disparities of their binocularly matched local features. This is not, however, always the case; the perceived depth of perceptual groupings can override local feature disparities (Wilde, 1950; Tausch, 1953; Ramachandran and Nelson, 1976). When this happens, an emergent 3D grouping can suppress “false matches” that are based on the real local disparities of their generative features in the outside world. The model can explain these data because the disparity filter is part of the 3D grouping process, lying as it does within the inhibitory interneurons in layer 2/3 V2 pale stripes, as well as data about how false binocular matches are suppressed that do not correspond to the objects in the outside world. The same perceptual grouping process can also simulate many other challenging data about 3D vision, including data about bistable perception and binocular rivalry (Grossberg and Swaminathan, 2004; Grossberg et al., 2008) and perceptual transparency and Kanizsa stratification (Grossberg and Yazdanbakhsh, 2005).

#### 1.2.6. 3D surface percepts form within filling-in-domains

An early model prediction (Grossberg, 1984) that boundary representations on their own do not give rise to visible percepts, indeed that “all boundaries are invisible,” is consistent with many perceptual and neurobiological data. This prediction is expected to hold at least within the interblob cortical stream between V1 and V4 in which perceptual boundaries are formed. In contrast, all visible percepts have been predicted to be a property of surface representations within the blob cortical stream from V1 to V4; see Grossberg (1994, 2014) for reviews. Boundaries are invisible, or amodal, because they pool opposite-polarity contrasts from both achromatic and chromatic receptive fields at the complex cell stage in the interblobs of cortical area V1 in order to build the best possible contrast-invariant boundaries of objects. In particular, they can build complete boundaries from objects that lie in front of textured backgrounds whose relative contrasts reverse along the object's perimeter (Figure 3B).

Surface representations are completed using a filling-in process that keeps opposite-polarity computations separate to enable distinct lightnesses and colors to fill-in within depth-selective Filling-In Domains, or FIDOs. This filling-in process can reconstruct lightness and color estimates in regions where they have been suppressed by the process of compensating for variable illumination, which is also called “discounting the illuminant” (Grossberg and Todorović, 1988). Boundaries control the depths at which different lightnesses and colors can fill-in via a process called 3D *surface capture*. The current simulations of the 3D LAMINART model illustrate only the filling-in of achromatic lightnesses in depth in response to psychophysical displays. Grossberg and Hong (2006) and Hong and Grossberg (2004), in their extension of 3D LAMINART to the Anchored Filling-In Lightness Model (aFILM) model, have simulated filling-in of surface lightnesses and colors in response to both psychophysical displays and natural scenes. Other articles have simulated surface percepts in response to artificial sensors such as Synthetic Aperture Radar (SAR), e.g., Grossberg and Williamson (1999) and Mingolla et al. (1999).

**Figure 5:**
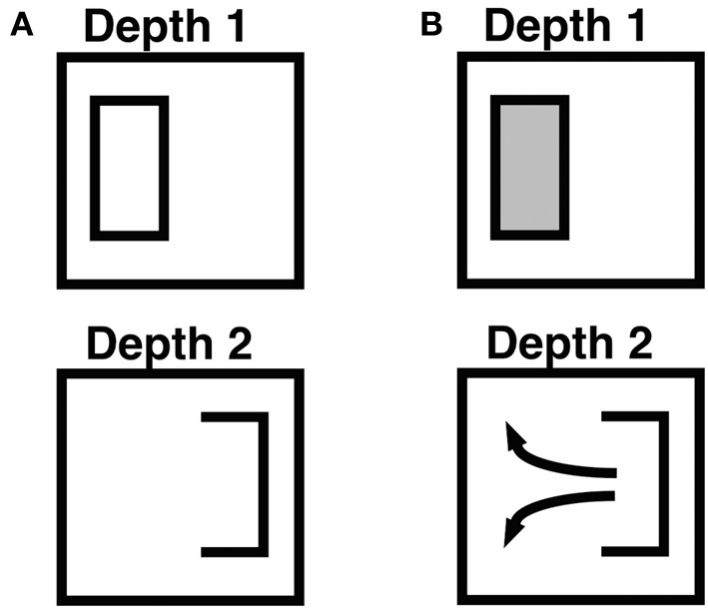
**Combining monocular and binocular boundaries, and filling-in within connected boundaries**. This figure illustrates how monocular and other depth-unselective boundaries, such as horizontal boundaries, are combined to control surface filling-in. The depicted boundaries, apart from the rectangular boundary frame, represent the boundaries that are induced by a part of an idealized da Vinci stereopsis display in which only the left vertical boundary within the frame is binocularly viewed. See the partially occluded window in Figure 2 of Grossberg (1994) for a complete da Vinci stereopsis display in which this kind of situation can occur. The right vertical boundary within the frame is viewed through only one eye, and is hence a monocular boundary that conveys no disparity information. The horizontal boundaries are processed in the same way as monocular boundaries. **(A)** Open and connected boundaries that are induced by the display after all boundaries are combined. The left vertical boundary is processed selectively at Depth 1 due to the binocular disparity that it induces. The right vertical boundary and the upper and lower horizontal boundaries are added to all boundary representations, across all depths, along the line of sight. This combination of boundaries creates a closed rectangular boundary at Depth 1 and an open boundary at Depth 2. These boundaries are projected topographically to the Filling-In DOmain, or FIDO, at the corresponding depth within which boundary-modulated filling-in of surface lightnesses and colors occurs. **(B)** Filling-in of surface lightness is contained or not depending on the connectedness of the boundary. At Depth 1, filling-in is contained within the closed rectangular boundary. At Depth 2, lightness can flow through the boundary gap and become uniform around both sides of the figure's boundary. As a result, the surface contour representation at Depth 2 generates no output signals via the contrast-sensitive network that outputs to subsequent processing stages. Reproduced with permission from Cao and Grossberg (2005).

How does the brain usually manage to fill-in lightnesses and colors at only the correct depths? The 3D LAMINART surface capture process is reviewed in Grossberg (1994), which also uses it to explain many data about 3D figure-ground perception. The 3D LAMINART model extends FACADE theory to laminar cortical circuitry, while also significantly expanding its explanatory and predictive range. A key surface capture property is that visible surfaces arise in cortical area V4 only if they are enclosed by *connected* boundaries. Figure 5 illustrates how, in response to a da Vinci stereopsis display, a rectangular connected boundary may be composed of one vertical binocular boundary that does encode disparity information, as well as one vertical monocularly viewed boundary and two horizontal boundaries that do not. Such a closed boundary can contain filling-in, and thereby support the formation of a visible surface percept. This surface percept will occur at the depth of the binocular boundary, if all other constraints are satisfied. At a different depth plane, where there is no vertical binocular boundary, however, then the total boundary contains a large gaps through which lightness and color signals can flow around the boundaries. Surface contrasts at the boundaries are hereby eliminated and, along with it, any filling-in of those surface features at higher processing stages.

#### 1.2.7. Surface-to-boundary feedback ensures perceptual consistency and initiates figure-ground separation

Boundaries and surfaces form according to computationally *complementary* rules: boundaries are completed *inwardly* using bipole cells, but surface filling-in spreads *outwardly*; boundaries form in an *oriented* way, but surface filling-in spreads *unoriented* in multiple directions; boundaries pool opposite-polarity contrasts at V1 complex cells and hereby become *insensitive to contrast polarity*, whereas visible surfaces are *sensitive to contrast polarity*. Given that boundaries and surface form using complementary computations, how do they typically work together to generate percepts whose perceived boundaries and surfaces are perceptually *consistent*? Otherwise expressed, how is *complementary consistency* achieved? Grossberg (1994) proposed that successfully filled-in regions within the surface representations send Surface-to-Boundary feedback to the boundary system (Figure 2) by sensing whether or not a surface region is filled-in within a connected boundary (see Figure 5B). Contrast-sensitive output circuits accomplish this by detecting where the bounding contours of a successfully filled-in region occur. Such boundary contours occur at positions where contrasts change quickly across space if the boundary can contain filling-in by being connected. If there are large gaps in a boundary that enable filling-in to spread out and around it, then there are no contrast differences around this boundary, so that no output signals are generated there.

These contrast-sensitive output signals are called *surface contours*. They are generated by on-center off-surround networks that operate within disparity and across position in response to filled-in surface activities. The surface contour outputs from the surface stream to the boundary stream strengthen, and thereby confirm, the boundaries that surround successfully filled-in surface regions, at the same time that they inhibit, or prune, redundant boundaries at the same positions and further depths (Grossberg, 1994, 1997; Grossberg and McLoughlin, 1997). The surface contour signals strengthen and prune their target boundaries by activating on-center off-surround networks that operate across disparity and within position within the boundary system. This boundary-enhancing property is predicted to interact with a developmental bias that favors the fixation plane. Taken together, these properties enable the simulation of stereopsis data that have not otherwise been explained; for example, see below and Cao and Grossberg (2005, 2012). Such a fixation plane bias may develop when the relatively high frequency of fixated percepts activates basic activity-dependent mechanisms of self-organizing maps whereby they enhance frequently activated cortical representations, e.g., Buonomano and Merzenich (1998).

Surface contour signals are also predicted to control where the eyes look and how the brain learns invariant object categories. In particular, because surface contour signals are strongest at the distinctive features of an attended object, they can be used to compute eye movement targets. The ARTSCAN, pARTSCAN, and ARTSCAN Search models (Fazl et al., 2009; Grossberg, 2009; Cao et al., 2011; Foley et al., 2012; Chang et al., 2014) predict how surface contour signals may generate predictive eye movement commands, via cortical area V3A, that (1) determine where the eyes will look next and (2) maintain spatial attention in posterior parietal cortex on an object's surface representation in V4, thereby (3) enabling inferotemporal cortex to learn view- and positionally-invariant object categories that represent its boundary and surface representations. Thus, the 3D LAMINART model is part of a larger architecture for active vision wherein 3D boundary and surface representations help to control eye movements for attending, seeing, searching, learning, and recognizing invariant object categories.

## 2. Model description

The 3D LAMINART model consists of two processing streams: a boundary stream and a surface stream (see Figures 1 and 2). The boundary stream runs from retina/LGN to V1 monocular and binocular boundaries and then to V2 binocular boundaries. The surface stream runs from retina/LGN to V1 and V2 monocular surfaces and then to V4 binocular surfaces. Figure 1 shows a laminar cortical circuit diagram of the 3D LAMINART model, and Figure 2 shows a functional block diagram of the model. A mathematical description of the model is provided in Section 4. In order to facilitate model understanding, the qualitative descriptions of model processes that is provided in this section also list the mathematical equation numbers in which these properties are rigorously defined in Section 4. In order to reduce computational load, the model currently simulates only horizontal and vertical contours and five boundary and surface depths. Five depths were chosen because they are enough to illustrate non-trivial depth separation. The model is extensible to any finite number of depths if finer depth discriminations are desired. Gradual changes of depth using a finite set of depth planes have also been simulated. In particular, Grossberg and Swaminathan (2004, Figure 23d) simulated slanted 3D percepts, including the Necker cube. Such continuous percepts of depth may be achieved by computing a weighted average of filled-in surface activities across multiple depth-selective filling-in domains.

Each model neuron is defined by membrane equation, or shunting, dynamics that have automatic gain control and contrast-normalization properties (Hodgkin, 1964; Grossberg, 1973, 1980). See Equations (1) and (2) in Section 4. Although model neurons and neurons *in vivo* will be clearly distinguished below, model neurons will be referred to by their neurophysiological names because their computational properties closely match those found in the brain. See Grossberg and Howe (2003), Grossberg and Yazdanbakhsh (2005), and Raizada and Grossberg (2003) for comparisons of model cell properties and connections with neurophysiological and neuroanatomical data.

### 2.1. Monocular boundaries

#### 2.1.1. LGN cells

The left and right retinal images are processed by LGN cells with circularly symmetric on-center off-surround receptive fields [see Figure 1A; variables *x*^*L/R*^_*ij*_ in Equations (3)–(5) in Section 4]. These LGN cells discount the illuminant and contrast-normalize the input scene.

#### 2.1.2. V1 monocular simple cells

LGN outputs activate monocular simple cells in layer 4 of the V1 interblobs. The simple cells are oriented filters (Hubel and Wiesel, 1968) that are sensitive to either a similarly oriented dark-light or light-dark contrast in the image, but not both [see Figure 1A; variables *s*^*L/R*^_*ijk*_ in Equations (6)–(8)]. Outputs from these simple cells project to both binocular simple cells and to monocular simple cells in Layer 3B [for the latter, see Figure 1A; variables *b*^*L/R*^_*ijk*_ in Equation (9)].

### 2.2. Binocular boundaries

#### 2.2.1. V1 binocular simple cells

Outputs from left and right eye monocular simple cells activate binocular simple cells in layer 3B of the interblobs, where steroscopic fusion begins [see Figure 1A; variables *b*^*B*^_*ijk*_ in Equation (10)]. The depth selectivity of binocular simple cells is determined by the retinal disparities of the layer 4 monocular cells that project to them. The binocular simple cells in layer 3B are sensitive to just one contrast polarity because only layer 4 simple cells with the same contrast polarity project to them (Figure 2A), thereby implementing the *same–sign hypothesis* at layer 3B simple cells that are selective for both binocular disparity and contrast polarity.

The selectivity of stereoscopic fusion at the binocular simple cells in layer 3B also requires the action of inhibitory interneurons [see Figure 1; variables *q*^*L/R*^_*ijkd*_ in Equations (11) and (12)]. The activity of a binocular simple cell is suppressed by these inhibitory interneurons if its left and right eye inputs differ too much in magnitude. The binocular simple cells hereby act like the “obligate cells” of Poggio (1991) by responding only when their left and right eye inputs are approximately equal in magnitude. These layer 3B obligate cells hereby help to solve the Correspondence Problem by responding only to stereoscopically fused stimuli with similar contrast amplitudes from the left and right eye retinal images.

The inhibitory interneurons ensure that binocular simple cells respond only to a narrow range of disparities by inhibiting each other through recurrent inhibitory interneurons, as well as their target binocular simple cell. These recurrent inhibitory interactions normalize total activity within the interneurons. Such normalization enables a “two-against-one” computation that leads to disparity selectivity, much as it leads to the bipole grouping property (see Section 1.2.5) during binocular boundary completion. V1 binocular simple cells and V2 binocular bipole cells thus both seem to use computationally homologous operations in different cortical regions and at different spatial scales. Future experiments should be designed to test this predicted homology and to clarify how it arises during brain evolution and development.

#### 2.2.2. V1 layer 2/3 monocular and binocular complex cells

Layer 2/3 contains complex cells that add inputs from simple cells at the same position that are sensitive to the same orientation but opposite contrast polarities. Both monocular complex cells [see Figure 1; variables *c*^*L/R*^_*ijk*_ in Equations (25) and (26)] and binocular complex cells [see Figure 1; variables *c*_*ijkd*_ in Equations (13)–(24)] are hereby formed. Because complex cells can respond to both contrast polarities, they can respond all along an object's boundary, even if contrast polarity with respect to the background reverses as its boundary is traversed (Figure 3B). Layer 2/3 complex cells thus implement an early cortical stage of contrast-invariant boundary detection. Complex cells also interact via long-range excitatory recurrent connections [variables *H*^*Ec*^_*ijkdv*_ in Equations (13) and (15)–(17)] and disynaptic inhibitory interneurons [variables *s*^*c*^_*ijkdv*_ in Equations (18)–(21)] that implement non-classical receptive fields via a bipole property (see Section 2.2.4). This, bipole property in V1 can only modulate a cell, not fire it, if the cell does not also receive a bottom-up input from simple cells.

#### 2.2.3. V2 layer 4 binocular cells

V1 layer 2/3 left and right monocular complex cells and binocular complex cells input to V2 layer 4 cells [see Figure 1; variables *v*_*ijkd*_ in Equations (27)–(29)]. The monocular cells, which do not compute a binocular disparity, are added to all depth planes in layer 4 along their respective lines-of-sight (Figure 5) as part of the brain's solution of the Monocular-Binocular Interface Problem. The layer 4 cells also receive surface contour feedback signals from left and right V2 monocular surfaces that are formed in the V2 thin stripe region [see Section 2.3.1 and Figure 1; variables *f*^*L*^_*ijkd*_, *f*^*R*^_*ijkd*_, and *f*_*ijkd*_ in Equations (28) and (29)] to help ensure perceptual consistency, despite the complementarity of boundary and surface computations. These surface contour surface-to-boundary feedback inputs modulate V2 layer 4 cells by enhancing their current level of activity. Surface contour feedback also helps to initiate figure-ground separation (Grossberg, 1994) and plays an indispensable role in explaining various 3D percepts such as da Vinci stereopsis (Cao and Grossberg, 2005).

#### 2.2.4. V2 layer 2/3 bipole grouping cells

Bipole grouping occurs among the binocular cells in V2 layer 2/3 [see Figure 1A; variables *g*_*ijkd*_ in Equations (30)–(35)]. These cells receive inputs from V2 layer 4 cells. These cells in layer 2/3 possess collinear, coaxial receptive fields that directly excite each other via long-range horizontal axons [variables *H*^*E*_*g*_^_*ijkdv*_ in Equations (30) and (32)]. They also give rise to short-range, disynaptic inhibitory interneurons [variables *s*^*g*^_*ijkdv*_ in Equation (34)] that inhibit themselves as well as their target complex cells [variables *H*^*Ig*^_*ijkd*_ in Equations (30) and (33)]. This balance of excitation and self-normalizing inhibition achieves the “two-against-one” bipole grouping property (Grossberg et al., 1997; Grossberg, 1999; Grossberg and Raizada, 2000; Grossberg and Williamson, 2001). The boundary grouping process, together with contrast-invariant boundary detection (Figures 1, 3B), allows well-localized and connected object boundaries to be completed even in response to disconnected and noisy boundary fragments.

Binocular cells in V1 layer 3B attempt to match every input from a vertical edge from one retinal image with nearby vertical edge input signals from the other retinal image within its disparity range, given that all the inputs code the same contrast polarity and approximately the same contrast magnitude. Even with these restrictions, there remains a Correspondence Problem because false matches may occur in V1.

Figure 4 illustrates the Correspondence Problem that may occur in response to four possible matches if each eye receives inputs from two bars. Only the two matches in the fixation plane are correct, and the other two are false. Such false matches are suppressed in V2 by the disparity filter [see DF in Figure 1; variables *G*^*P*^_*ijkd*_ are the total DF inhibition in Equations (30) and (35)] as part of the bipole grouping process. The model's disparity filter (solid lines between Figure 4 neurons) encourages unique matching using inhibition between neurons that share a monocular input. These disparity filter inhibitory interactions are, in addition, symmetrical about the fixation plane with the fixation plane inhibiting the near and far disparity planes more than conversely (see **Table 2**). The line-of-sight inhibition and the fixation plane advantage together select two matches in the fixation plane, thereby helping to solve the Correspondence Problem. Because disparity filter interactions occur among the inhibitory interactions that control perceptual grouping in V2 layer 2/3, the model parsimoniously combines suppression of false matches with long-range Gestalt grouping processes.

### 2.3. Monocular surfaces

#### 2.3.1. V2 monocular surfaces

Monocular surfaces are computed in the model V2 thin stripes [see Figure 1; variables *F*^*L/R*^_*ijd*_ in Equations (36)–(45)]. The left (right) V2 thin stripe receives boundary signals from V2 layer 2/3 complex cells and illumination-discounted lightness signals from left (right) LGN cells via the left (right) V1 blob region (see Figure 1). Earlier FACADE theory and 3D LAMINART modeling has predicted and simulated how surface representations may be generated by a filling-in process, whether through nearest-neighbor diffusive interactions (Cohen and Grossberg, 1984; Grossberg and Todorović, 1988; Grossberg, 1994) or long-range horizontal connections (Grossberg and Hong, 2006). Psychophysical data (e.g., Paradiso and Nakayama, 1991; Pessoa and Neumann, 1998; Pessoa et al., 1998) and neurophysiological data (e.g., Rossi et al., 1996; Lamme et al., 1999) support the existence of filling-in, in contradiction of the critique of Dennett (1991) that such a process does not exist. Surface filling-in has, in fact, been used to explain and simulate many percepts that have not been explained without it, such as surface percepts during figure-ground separation (Kelly and Grossberg, 2000); 2D and 3D neon color spreading and transparency (Grossberg and Mingolla, 1985a; Grossberg, 1994; Grossberg and Yazdanbakhsh, 2005); 3D shape-from-texture (Grossberg et al., 2007); bistable 3D percepts such as the Necker cube illusion (Grossberg and Swaminathan, 2004); and multiple lightness and color percepts (Grossberg and Todorović, 1988; Grossberg and Kelly, 1999; Hong and Grossberg, 2004; Grossberg and Hong, 2006). As illustrated by Figure 5, a consciously perceived surface representation can rise from filling-in only if it is enclosed by a connected boundary.

The LGN cells interact through on–center, off–surround circularly symmetric receptive fields whose interactions discount the effects of spatially non-uniform illumination; that is, “discount the illuminant.” These excitatory and inhibitory interactions are balanced and thus attenuate cell activity in response to spatially uniform or slowly varying stimulation. Model LGN cells hereby respond preferentially to luminance borders. At later filling-in stages, these illuminant-discounted surface-border, also called feature contour, signals propagate across surface regions that are enclosed by connected boundaries to complete the lightness representation. Early simulations of filling-in used a boundary-gated nearest-neighbor diffusion equation (Grossberg and Todorović, 1988). Connected boundaries from V2 layer 2/3 create resistive barriers to limit signal spread (Figure 5) of the lightness signals received from the LGN within the V2 thin stripes. Grossberg and Hong (2006) have simulated how filling-in can be carried out by boundary-gated long-range horizontal interactions that operate 1000 times faster than diffusion. An interesting open problem concerns how long-range excitatory interactions may develop, on the one hand, to control the inward and oriented properties of bipole grouping and, on the other hand, to control the complementary outward and unoriented properties of surface filling-in.

The monocular surfaces that are formed in V2 thin stripes are predicted to be invisible, or amodal, and thus do not subserve visible 3D surface percepts. They can, however, directly activate pathways leading to their amodal recognition within the inferotemporal cortex (Grossberg, 1994).

### 2.4. Surface-to-boundary feedback signals

Successfully filled-in monocular surfaces send surface contour signals from positions at which filled-in contrast changes rapidly enough across space into the boundary representations via V2 layer 4 [see Figures 1, 2, Section 1.2.7, and variables *f*^*L/R*^_*ijkd*_ in Equations (46)–(48)]. These surface-to-boundary feedback signals modulate the activities of V2 boundary cells so that the boundaries that surround the successfully filled-in surfaces are enhanced and redundant boundaries are suppressed, thereby ensuring perceptual consistency and contributing to figure-ground separation

### 2.5. Binocular surfaces

#### 2.5.1. V4 surfaces

Consciously visible binocular 3D surface percepts are generated in cortical area V4, where 3D figure-ground separation of object surfaces is also predicted to be completed [see Figure 1; variables *w*_*ijd*_ in Equations (49) and (50)]. Area V4 receives boundary signals from V2 layer 2/3 and lightness signals from the LGN via V1 blobs and V2 thin stripes. The surface filling-in process is similar to the one described in Section 2.3, except V4 combines monocular lightness signals from both eyes.

The current model simplifies the V4 interactions that have been predicted in Grossberg (1994) to generate 3D surface percepts. See Grossberg and Swaminathan (2004) and Grossberg and Yazdanbakhsh (2005) for simulations of the additional boundary and surface interactions needed to explain percepts that involve the separation of overlapping figures from their backgrounds, such as the Necker cube and 3D neon color spreading.

## 3. Model simulations

This section summarizes a simulation of the Venetian blind effect and of Panum's limiting case to illustrate how monocular and binocular information may interact in the laminar circuits of visual cortex to generate 3D surface percepts. For the Venetian blind simulation, each eye's stimulus was presented on a grid 30 units high and 115 units wide. For the Panum's limiting case stimulus, each eye's stimulus was presented on a grid 30 units high and 60 units wide, which was sufficient to process the smaller width of the stimulus. In all simulations, the background had a luminance value, in arbitrary units, of 2. In the simulation figures, the light gray bars (if any) had a luminance of 1 and the dark gray bars 0.1. Simulations were performed using the *Matlab* software package.

### 3.1. The venetian blind effect

**Figure 6:**
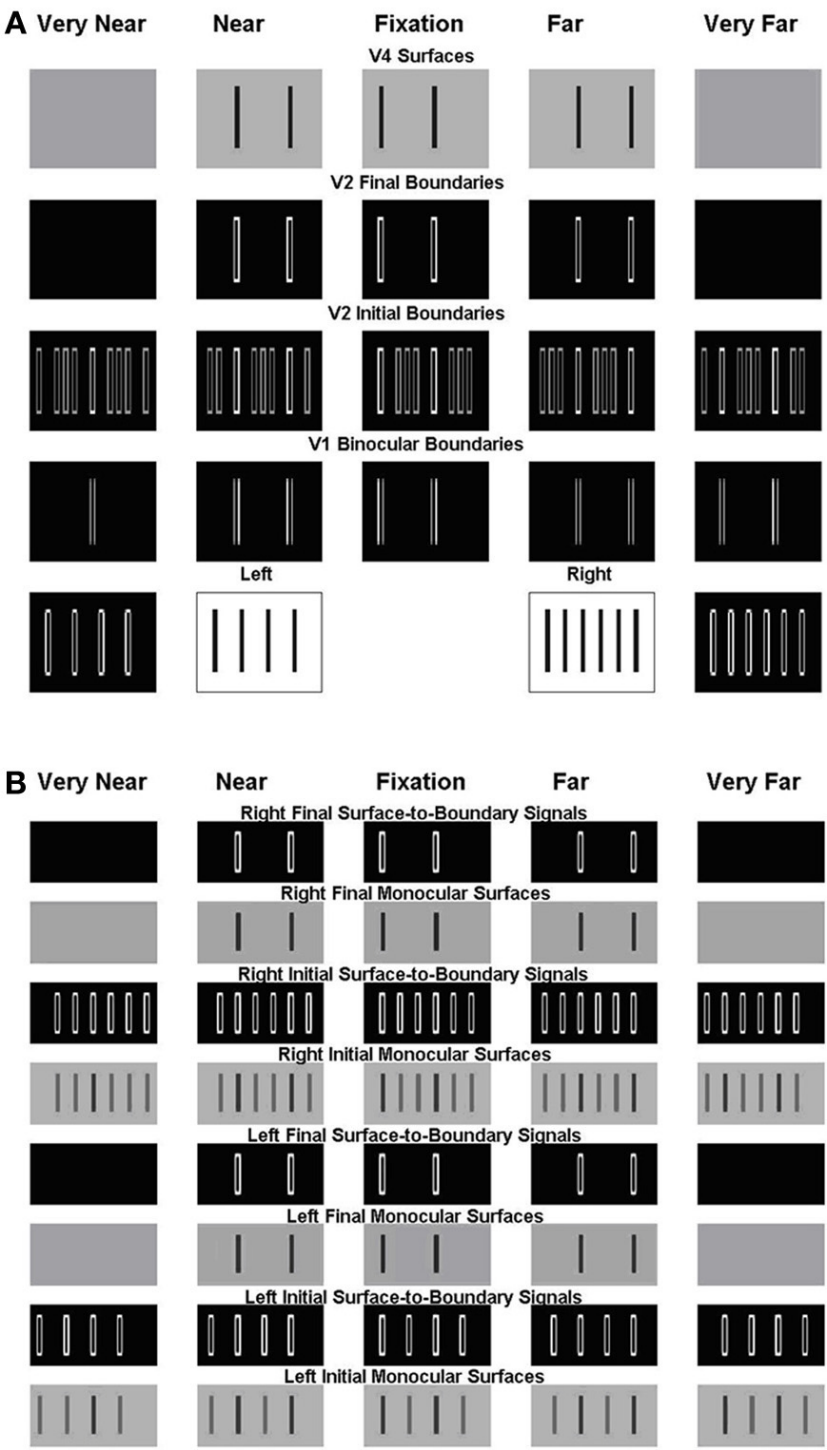
**Simulation of the Venetian blind effect in Howard and Rogers (1995)**. The variables that are plotted in this and other simulations correspond to the model processing stages shown in Figure 2, and the corresponding mathematical variables shown in Figure 1. **(A)** The two middle plots of the bottom row are the Left Eye Inputs and the Right Eye Inputs, respectively. The two outer plots in the bottom row are the Left Monocular Boundaries that are computed at the Left Eye V1 Monocular Complex Cells and the Right Monocular Boundaries that are computed at the Right Eye V1 Monocular Complex Cells, respectively. The plots in the third row from the bottom are the Initial Binocular Boundaries that are computed in V2 Layer 4. The plots in the fourth row from the bottom are the Final Binocular Boundaries that are computed in V2 layer 2/3. The initial boundaries in V2 layer 4 are computed before the Surface-to-Boundary feedback signals act (see S-to-B in Figure 2). The final boundaries are computed after all the boundaries equilibrate to the Surface-to-Boundary feedback signals. The plots in the top row are the V4 Binocular Surfaces. **(B)** These simulation figures show the Left and Right, Initial and Final, Monocular Surfaces and Surface-to-Boundary Signals. As in **(A)**, Initial values are computed before the Surface-to-Boundary feedback signals act. Final values are computed after all the surfaces equilibrate to the Surface-to-Boundary feedback signals. See text for further details.

Section 1.1 described a Venetian blind stereogram from Figure 6.21 of Howard and Rogers (1995). This display consists of a low frequency grating that is presented to the left eye, and a high frequency presented to the right eye. Every second bar of the left grating is in retinal correspondence with every third bar of the right grating; see the middle two plots of the first row of Figure 6A.

Figure 6 includes two parts: Figure 6A V1 and V2 boundaries and V4 surfaces and Figure 6B V2 monocular surfaces and surface-to-boundary feedback signals. Like the model diagram shown in Figures 1A, 6A should be read from the bottom up, with the bottom two rows representing the input (inner pair of figures with black bars on white background) and the V1 boundary representations (white boundaries on black background), the next two rows representing the V2 boundary representations before (V2 Initial Boundaries) and after (V2 Final Boundaries) surface-to-boundary feedback acts, and the top row representing the V4 surface representations. In Figure 6B, the bottom four rows represent, respectively, in ascending order, the following quantities corresponding to the left eye: the Initial Monocular Surfaces before any feedback interactions occur, the Initial Surface-to-Boundary Signals generated by these surfaces, the Left Final Monocular Surfaces after the Surface-to-Boundary Signals have their effect, and the Final Surface-to-Boundary Signals that are caused by the Final Monocular Surfaces. The same quantities are represented for the right eye in the top four rows. In the top four rows of Figure 6A and all rows of Figure 6B, depth increases from left to right. The middle plot representing the fixation plane. The two leftmost plots represent the two near depth planes. Finally, the two right plots represent the two far depth planes.

This stereogram induces a percept of short ramps, each containing three bars. The bars slope up from left to right and are separated by step returns. The model correctly simulates this surface percept; see the top row of Figure 6A, which shows the simulated depthful surface representations in the model area V4. This row shows that, reading positions from left to right, the first bar of the percept is in the zero disparity plane (fixation plane; second column), the second in the near disparity plane (first column), then there is a step return to the third bar which is located in the far disparity plane (third column), after which the pattern repeats.

**Figure 7:**
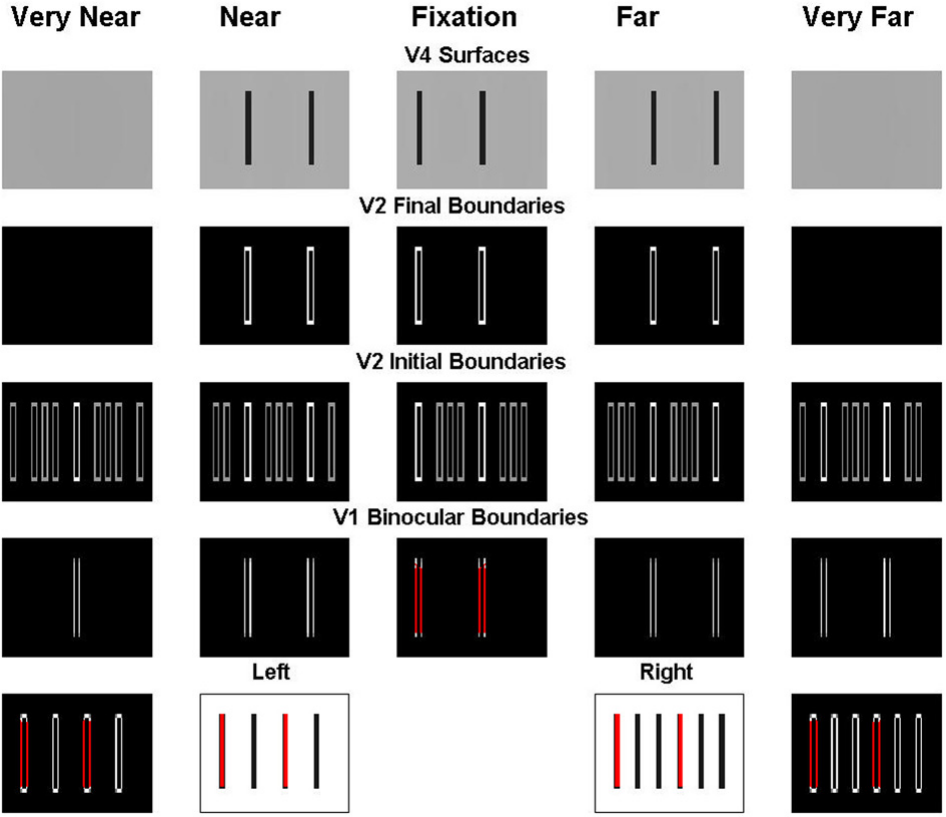
**Explanation of the simulation of the Venetian blind effect where the bars in retinal correspondence and their binocular fusion in the fixation plane are marked in red**. See the caption of Figure 6 for an explanation of the variables being simulated, and the text for further details.

Note that every second bar of the left input is in retinal correspondence with every third bar of the right input (bottom row). In Figure 7, these bars are marked in red color (the middle two plots of the bottom row) as are their monocular vertical boundaries (the left most and right most plots of the bottom row). Because of the retinal correspondence, their vertical boundaries are matched in the fixation plane by V1 binocular cells, marked in red color in the middle plot of the second row (reading from the bottom). According to the advantage of the fixation plane (Section 2.2.4), every vertical boundary located in the same line-of-sight of these binocularly matched vertical boundaries will be killed by the line-of-sight inhibition, whether binocularly matched or not. In particular, vertical boundaries in the very near and very far depths (the first plot and the last plot of the second row) will all be killed. But the binocularly matched vertical boundaries in the near and far depths (the second plot and the fourth plot of the second row) are not in the same lines-of-sight of the binocular boundaries in the fixation plane, and hence survive the inhibition from them.

**Figure 8:**
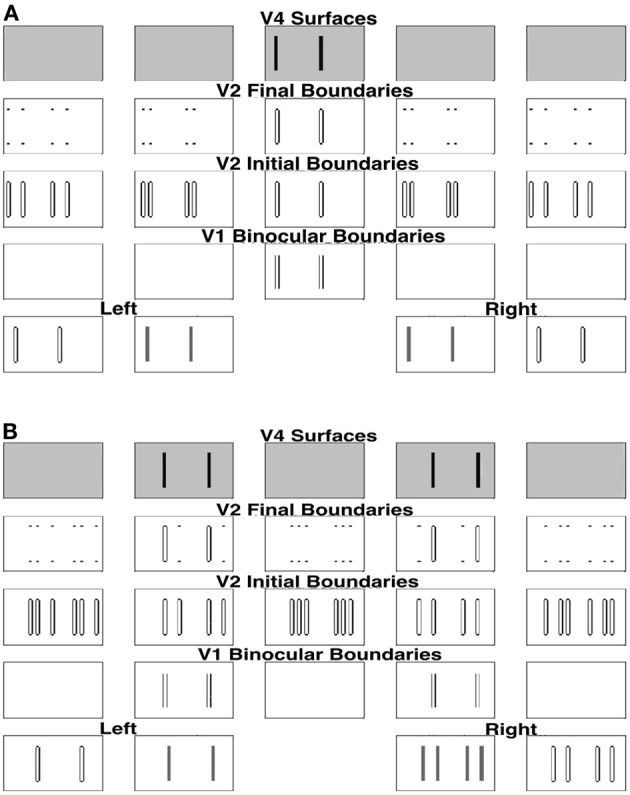
**(A)** Simulation of one component of the Venetian blind effect. **(B)** Simulation of the other component. See the caption of Figure 6 for an explanation of the variables being simulated, and the text for further details.

**Figure 9:**
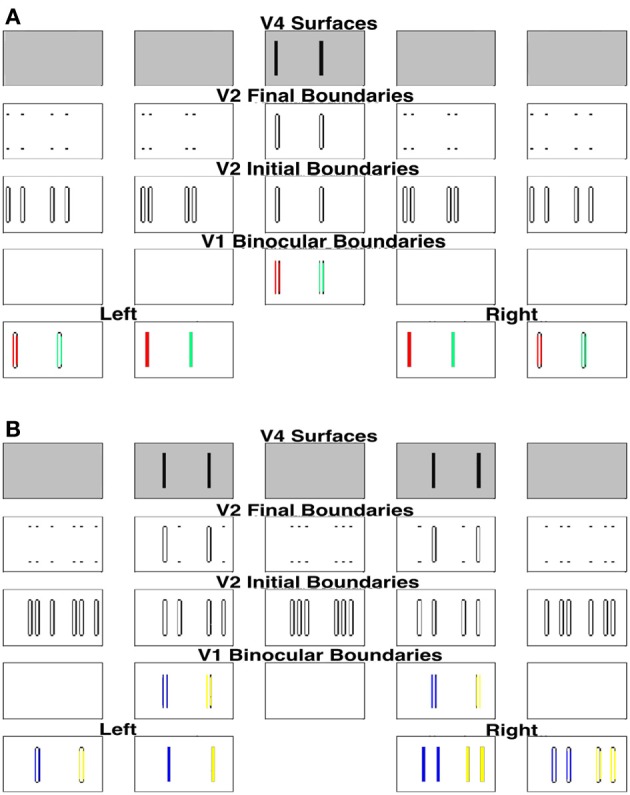
**Explanation of the simulation of components of the Venetian blind effect where the binocular fused bars are marked in the same color**. **(A)** Processing of image components in retinal correspondence. **(B)** Processing of image components that are disparate in the two eyes.

In order to simplify the explanation, we can divide the stimulus into two components and consider these separately as follows. First, we extract the bars in retinal correspondence to form the stimulus shown in the middle two plots of the first row of Figure 8A. Since the bars in the left and right inputs of this figure are in retinal correspondence, the model correctly predicts that they will appear in the fixation plane, as shown by the middle plot of the fifth row. The specific binocular matches are shown in Figure 9A, where the matched bars are marked in the same color.

For the remaining bars, shown in Figure 8B, the right eye sees twice the number of bars as the left eye, as in Panum's limiting case, which is explained in the next section. The model simulates fusion of each bar of the left input with two bars of the right input to induce the percept shown in the top row of Figure 8B. Figure 8B shows how these bars are fused, where the fused bars are marked in the same color and form two side-by-side Panum's limiting cases. The binocular matches are shown in Figure 9B.

Adding together the percepts shown in top rows of Figures 8A,B yield the surface percept shown in the top row of Figure 6A, thereby explaining this Venetian blind effect. Figure 6B shows the left and right V2 monocular surfaces and the corresponding surface-to-boundary signals, in both initial and final stages. The surface-to-boundary signals enhance the surviving boundaries with a closed contour that contains surface filling-in, and therefore helps to generate the correct percept.

The insight that the model provides is that this example of the Venetian blind effect is just a complex version of the Correspondence Problem as it is illustrated by Panum's limiting case, when it is properly understood by combining early stereo matching, later boundary selection by a disparity filter that is part of the boundary grouping process, and surface filling-in of those regions that are completely enclosed by connected boundaries.

### 3.2. Dichoptic masking in Panum's limiting case

**Figure 10:**
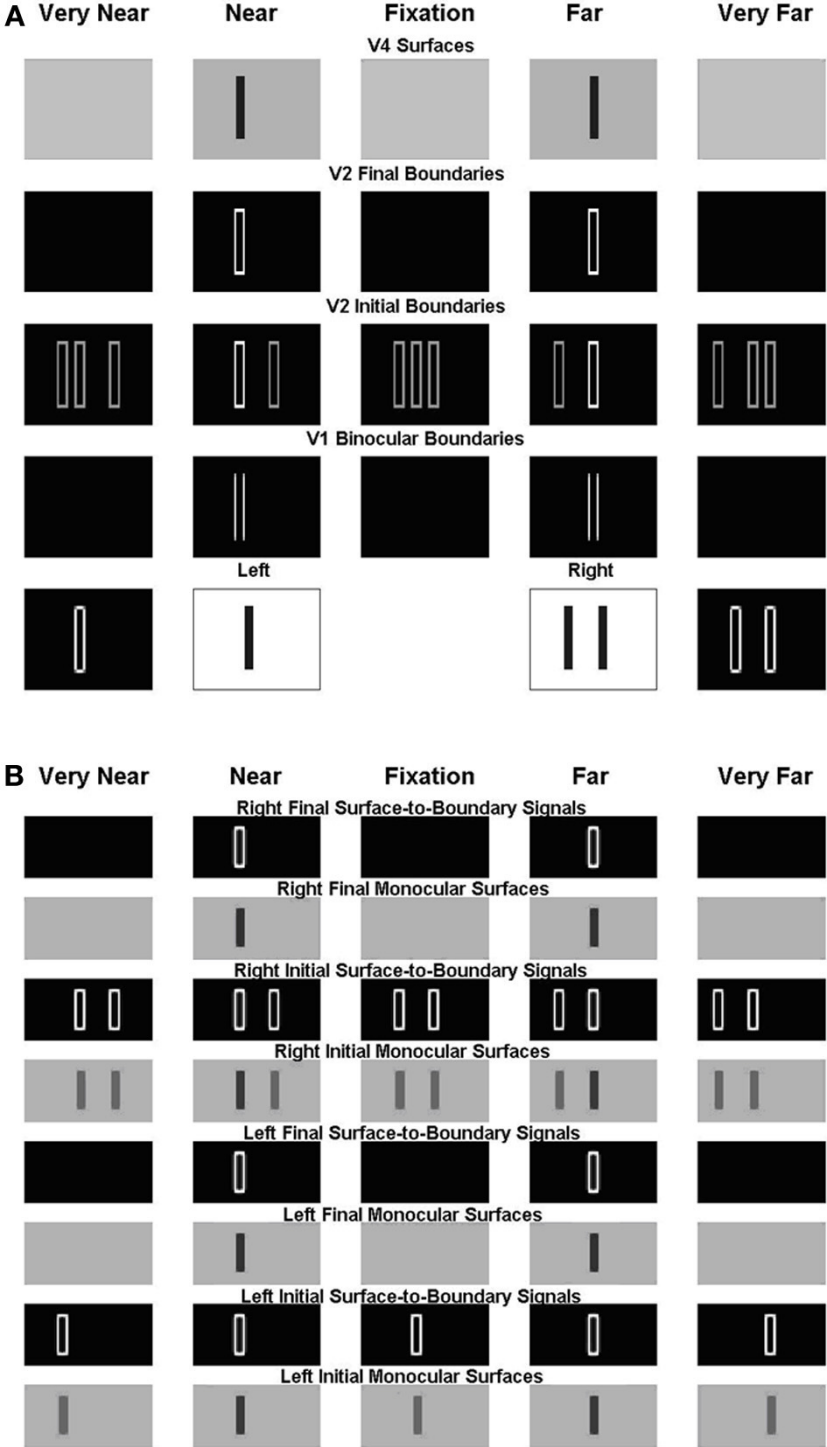
**Simulation of dichoptic masking in Panum's limiting case studied by McKee et al. (1995)**. See the caption of Figure 6 for an explanation of the variables being simulated, and the text for further details.

As described in the previous section, the present model solves the Correspondence Problem by using a disparity filter that encourages unique matching, via line-of-sight inhibition, but does not enforce it. One advantage of this is that the model can simulate Panum's limiting case, where a bar in one eye is simultaneously fused with two bars in the other eye (Panum, 1858; Gillam et al., 1995; McKee et al., 1995). Figure 10 shows the model simulation where a bar in one eye masks equally two bars presented to the other eye as reported by McKee et al. (1995).

For this stimulus, the left eye receives a single bar input, whereas the right eye receives an input consisting of two bars; see the middle two plots within the first row of Figure 10A. The monocular boundaries that are induced by these stimuli are shown within the outer two plots of the first row. The bar of the left input fuses with both bars of the right input at V1 binocular boundary cells, thereby forming binocular boundaries in both a near and a far disparity plane; see, the second and fourth plots of the second row. In V2, monocular boundaries are added to all disparity planes along their respective lines-of-sight; see the third row. Left monocular boundaries induce the left bar representation in the first two plots, the middle bar representation in the third plot, and the right bar representation in the fourth and fifth plots of the third row. Right monocular boundaries induce the two right bar representations in the first two plots, the outer two bar representations in the third plot, and the leftmost two bar representations in the fourth and fifth plots of the third row. The V1 monocular and binocular boundaries are added within V2. V2 boundaries that combine binocular input and monocular inputs are stronger than those that do not. Recurrent inhibition of the V2 disparity filter suppresses the V2 vertical boundaries that receive only monocular input and that share one of their lines-of-sight. The binocularly matched boundaries in the near and far depths, represented, respectively, in the third row by the first bar of the second plot and the second bar of third plot, are in the same line-of-sight. But they have the same (or almost the same) strength, and therefore do not kill each other because the disparity filter encourages unique matching, via line-of-sight inhibition, but does not enforce it. The surviving V2 boundary representations are shown in the fourth row. Those regions in V2 that are enclosed by a connected boundary give rise to surface percepts in V4, as shown in the fifth row.

Figure 10B shows the left and right V2 monocular surfaces and the corresponding surface-to-boundary signals, in both initial and final stages. The surface-to-boundary signals enhance the surviving binocular boundaries in the second and fourth plots of the fourth row of Figure 9A, which have closed contours that enclose the surface filling-in process, and therefore generate the correct surface percept. The model correctly predicts that the bar of the left input is matched with both bars of the right input, and so masks them both equally (McKee et al., 1995).

## 4. Model equations

This section describes the 3D LAMINART model equations. Since it describes the model used in Cao and Grossberg (2005), the description of the equations is adapted from the exposition in that article.

Each neuron is a single voltage compartment whose membrane potential, *v*(*t*), obeys:

(1) $$\begin{array}{l} C_{m}\frac{dv(t)}{dt}=(E_{leak}-v(t))g_{leak}+(E_{excit}-v(t))g_{excit}(t)\\ \;+(E_{inhib}-v(t))g_{inhib}(t). \end{array}$$

In (1), parameters *E* denote reversal potentials, g_*leak*_ is a constant leakage conductance, and the time-varying conductances *g*_*excit*_(*t*) and *g*_*inhib*_(*t*) are the total excitatory and inhibitory inputs to the cell (Hodgkin, 1964; Grossberg, 1968, 1973). The products of potential differences times conductances define shunting interactions that enable automatic gain control and normalization of activity to occur among the interacting variables. The following notation is used for the capacitance term *C*_*m*_ = 1, the leakage conductance *g*_*leak*_ = *A*, and the reversal potentials *E*_*excit*_ = *B, E*_*inhib*_ = −*C*, and *E*_*leak*_ = 0. When Equation (1) can be rewritten as:

(2) $$\frac{dv}{dt}=-Av+(B-v)g_{excit}-(C+v)g_{inhib}.$$

In (2), *A* is a constant decay rate, *B* is the maximum membrane potential, *C* is the minimum membrane potential, *g*_*excit*_ is the total excitatory input, and *g*_*inhib*_ is the total inhibitory input. All the simulations use a single set of parameters. For simplicity, the same parameter symbol (e.g., *α*) may be used in different equations, but with a different value, that is specified in each equation Figure 1 labels the model processing stages with the corresponding mathematical variable names to facilitate tracking the network relationships among the various variables. The non-technical summary of model processes in Section 2 parallels the definition of mathematical equations in this section to provide further expository support. Simulations were performed using *Matlab*.

### 4.1. LGN

The LGN cell membrane potentials, *x*^*L/R*^_*ij*_, obey the following shunting on-center off-surround equation:

(3) $$\frac{dx_{ij}^{L/R}}{dt}=-\alpha x_{ij}^{L/R}+\left(\beta -x_{ij}^{L/R}\right)I_{ij}^{L/R}-x_{ij}^{L/R}\sum_{p\neq i,q\neq j}G_{pqij}I_{pq}^{L/R},$$

where L/R designates that the cell belongs to the left or right monocular pathway, indices i and j denote the position of the input on the retina, *α* is a constant (10^−5^) rate of decay, β is a constant (9.9) maximum membrane potential, *I*^*L/R*^_*ij*_ is the luminance input of the left or right retinal image to the excitatory on-center, and *G*_*pqij*_ is a Gaussian inhibitory off-surround kernel:

(4) $$G_{pqij}=\exp\left(-\frac{{\left(p-i\right)}^{2}+{\left(q-j\right)}^{2}}{2\sigma^{2}}\right),$$

where σ scales the kernel size (1.5). The steady-state cell membrane potentials are:

(5) $$x_{ij}^{L/R}=\frac{\beta I_{ij}^{L/R}}{\alpha +\sum_{p,q}^{}G_{pqij}I_{pq}^{L/R}}.$$

Equation (5) was used in the simulations.

### 4.2. V1 layer 4 simple cells

At steady-state, the membrane potentials, *s*^*L/R*,+^_*ijk*_, of simple cells that respond to dark-light contrast polarity are given by:

(6) $$s_{ijk}^{L/R,+}=\sum_{p,q}{\text{K}}_{pqk}^{}{\left[x_{i+p,j+q}^{L/R}\right]}^{+}.$$

In (6), the superscript *L/R*, + means *L*+ or *R*+. *L* denotes left monocular, *R* denotes right monocular, + indicates that the simple cell responds to dark-light contrast polarity, and *k* denotes orientation. Two orientations, vertical (*k* = 1) and horizontal (*k* = 2), were simulated. The threshold linear signal function [x]^+^ = max(x,0), and K_*pqk*_ is an orientationally-tuned Gabor kernel:

(7) $$\begin{array}{l} {\text{K}}_{pqk}^{}=\phi \sin\left(\frac{2\pi (r-0.5)}{\tau }\right)\\ \;\exp\left[-\frac{1}{2}\left(\frac{{\left(p-0.5\right)}^{2}}{\sigma_{p}^{2}}+\frac{{\left(q-0.5\right)}^{2}}{\sigma_{q}^{2}}\right)\right]\text{​}. \end{array}$$

In (7), terms ϕ, τ, σ_*p*_, σ_*q*_ are constants (4.4, 3π, 0.6, 0.6) representing kernel amplitude and dimensions; *r* = *p* for cells that respond to vertical boundaries; and *r* = *q* for those that respond to horizontal boundaries.

The cell membrane potentials of simple cells with light–dark contrast polarity are the inverse of those in (6):

(8) $$s_{ijk}^{L/R,-}=-s_{ijk}^{L/R,+}=-\sum_{p,q}{\text{K}}_{pqk}^{}{\left[x_{i+p,j+q}^{L/R}\right]}^{+}.$$

### 4.3. V1 layer 3B monocular simple cells

The steady-state membrane potentials, *b*^*L/R*,+/−^_*ijk*_ of the layer 3B monocular simple cells obey:

(9) $$b_{ijk}^{L/R,+/-}=2{\left[s_{ijk}^{L/R,+/-}\right]}^{+}.$$

The factor of 2 compensates for monocular simple cells receiving an input from one eye whereas binocular simple cells receive inputs from both eyes.

### 4.4. V1 layer 3B binocular simple cells

The layer 3B binocular simple cells implement the obligate property by receiving excitatory inputs from layer 4 and balanced inhibitory input from layer 3B inhibitory interneurons at the same position and disparity. Their membrane potentials, *b*^*B*,+/−^_*ijkd*_ obey:

(10) $$\begin{array}{l} \frac{d}{dt}b_{ijkd}^{B,+/-}=-\gamma_{1}b_{ijkd}^{B,+/-}+\left(1-b_{ijkd}^{B,+/-}\right)\\ \;\left({\left[s_{(i+s)jk}^{L,+/-}-\theta \right]}^{+}+{\left[s_{(i-s)jk}^{R,+/-}-\theta \right]}^{+}\right)\\ \;-\alpha ({\left[q_{ijkd}^{L,+/-}\right]}^{+}+{\left[q_{ijkd}^{L,-/+}\right]}^{+}\\ \;+{\left[q_{ijkd}^{R,+/-}\right]}^{+}+{\left[q_{ijkd}^{R,-/+}\right]}^{+}), \end{array}$$

where the parameters γ_1_, α, and θ (0.1, 7.2, 0.4) represent the decay rate of the membrane potential, the inhibitory gain, and the signal threshold. The variables *q*^*L/R*,+/−^_*ijkd*_ are membrane potentials of inhibitory interneurons in layer 3B, *k* is the orientation, *d* is the disparity to which the model neuron selectively fires, and index *s* is the positional shift between left and right eye inputs that depends on the disparity; see Table 1.

**Table 1:** **The allelotropic shift (*s*) is the amount that the left and right monocular contours must be displaced to form a single fused binocular contour**.

**Disparity (*d*)**	**V. Near disparity**	**Near disparity**	**Zero disparity**	**Far disparity**	**V. Far disparity**
**Allelotropic shift (*s*)**	−8	−4	0	+4	+8

*It depends on the disparity. It is zero for matches in the fixation plane because these matches are between contours at retinal correspondence. Figure 3 illustrates the allelotropic shift and shows that a left monocular contour needs to be shifted more to the right for matches that are further from the observer, whereas a right monocular contour needs to be shifted in the opposite direction*.

Layer 3B inhibitory interneuronal cell membrane potentials, *q*^*L/R*,+/−^_*ijkd*_, receive excitatory input from layer 4 and inhibitory input from all other inhibitory interneurons that code the same position and disparity. Their left (L) and right (R) subpopulations obey:

(11) $$\begin{array}{l} \frac{d}{dt}q_{ijkd}^{L,+/-}=-\gamma_{2}q_{ijkd}^{L,+/-}+{\left[s_{(i+s)jk}^{L,+/-}-\theta \right]}^{+}-\beta ({\left[q_{ijkd}^{R,+/-}\right]}^{+}\\ \;+{\left[q_{ijkd}^{R,-/+}\right]}^{+}+{\left[q_{ijkd}^{L,-/+}\right]}^{+}), \end{array}$$

and

(12) $$\begin{array}{l} \frac{d}{dt}q_{ijkd}^{R,+/-}=-\gamma_{2}q_{ijkd}^{R,+/-}+{\left[s_{(i-s)jk}^{R,+/-}-\theta \right]}^{+}-\beta ({\left[q_{ijkd}^{L,+/-}\right]}^{+}\\ \;+{\left[q_{ijkd}^{L,-/+}\right]}^{+}+{\left[q_{ijkd}^{R,-/+}\right]}^{+}). \end{array}$$

In (11) and (12), the parameters γ_2_, β, and θ (4.5, 4, 0.4) are the membrane potential decay rate, the inhibitory gain, and the signal threshold, respectively.

Mild constraints on these parameter values are sufficient to ensure that the binocular simple cells act like “obligate cells” (Poggio, 1991) that respond vigorously only when their left and right inputs are approximately equal in size. Equation (10) was solved at equilibrium, using the equations described in the Obligate Theorem (see Section 4) to speed up the simulations.

### 4.5. V1 layer 2/3 monocular and binocular complex cells

V1 layer 2/3 monocular and binocular complex cells pool the cell membrane potentials of monocular and binocular layer 3B simple cells of both contrast polarities at each position and orientation. These complex pyramidal cells also emit long-range, collinear, coaxial connections within layer 2/3 whereby they excite each other. The long-range excitatory connections also diverge to excite short-range, disynaptic interneurons that inhibit target complex cells and nearby inhibitory interneurons. The recurrent inhibition normalizes the total activity of the inhibitory interneuronal network. The “two-against-one” balance of excitation and inhibition that converges on the pyramidal cells implements the bipole property that controls boundary grouping (Grossberg and Mingolla, 1985a,b; Grossberg and Raizada, 2000). Monocular and binocular bipole cells obey the same laws but have different inputs. The membrane potential, *c*_*ijkd*_, of binocular collinear bipole cell in V1 layer 2/3 obeys:

(13) $$\begin{array}{l} \frac{d}{dt}c_{ijkd}=-\alpha c_{ijkd}+\left(\beta -c_{ijkd}\right)\\ \;\left(I_{ijkd}^{c}\left(\gamma_{1}+\gamma_{2}{\left[\sum_{v}H_{ijkdv}^{E_{c}}-H_{ijkd}^{I_{c}}\right]}^{+}\right)+\gamma_{3}{\left[c_{ijkd}-\beta_{c}\right]}^{+}\right)\\ \;-\left(1+c_{ijkd}\right)\left(C_{ijkd}^{P}+C_{ijkd}^{S}\right), \end{array}$$

with parameters α, β, γ (α = 20, β = 7, γ_1_ = 1, γ_2_ = 1, γ_3_ = 0.5). The input *I*^*c*^_*ijkd*_ from V1 layer 3B binocular simple cells obeys:

(14) $$I_{ijkd}^{c}=\mu \left({\left[b_{ijkd}^{B,+}-\theta \right]}^{+}+{\left[b_{ijkd}^{B,-}-\theta \right]}^{+}\right).$$

with parameters μ and θ (20, 0.1). The excitatory input *H*^*Ec*^_*ijkdv*_ derives from long-range recurrent connections in V1 layer 2/3 to a complex cell at position (*i,j*), orientation *k*, disparity *d*, and side *v* of the bipole cell (Grossberg and Swaminathan, 2004). Term ∑_*v*_*H*^*E*_*c*_^_*ijkdv*_ sums inputs from both sides *v* = 1, 2 of the bipole cell:

(15) $$H_{ijkdv}^{Ec}=\sum_{pq}W_{pqijkv}^{c}{\left[c_{pqkd}-\zeta_{c}\right]}^{+}.$$

In (15), ζ_*c*_ is a threshold (0), and *W*^*c*^_*pqijkv*_ is the long-range connection weight from the bipole cell at position (*p,q*) to the bipole cell at position (*i,j*), orientation *k*, and side *v*. The connection weights for the horizontal orientation (*k* = 2) obey (*v = 1* for left branch and *v* = 2 for right branch):

(16) $$W_{pqij21}^{c}={\left[sign(i-p)\exp\left(-\left(\frac{{\left(i-p\right)}^{2}}{\sigma_{p}^{2}}+\frac{{\left(j-q\right)}^{2}}{\sigma_{q}^{2}}\right)\right)\right]}^{+}\text{​​},$$

and

(17) $$W_{pqij22}^{c}={\left[sign(p-i)\exp\left(-\left(\frac{{\left(i-p\right)}^{2}}{\sigma_{p}^{2}}+\frac{{\left(j-q\right)}^{2}}{\sigma_{q}^{2}}\right)\right)\right]}^{+}\text{​​},$$

where *sign(x)* = 1 if *x* > 0, −1 if *x* < 0, and 0 otherwise. Parameters σ_*p*_ = 8, σ_*q*_ = 0.3, and the spatial connection range (diameter) is 3. Connection weights for the vertical orientation are obtained by rotation.

Term *H*^*Ic*^_*ijkd*_ is the total inhibitory input from the inhibitory interneurons:

(18) $$H_{ijkd}^{Ic}=\sum_{v}{\left[s_{ijkdv}^{c}\right]}^{+}.$$

In (18), *s*^*c*^_*ijkdv*_ is the activity of the interneuron that inhibits the bipole cell at position *(i,j)*, orientation *k*, disparity *d*, from side *v*, where:

(19) $$\frac{d}{dt}s_{ijkdv}^{c}=\delta_{I}\left(-s_{ijkdv}^{c}+H_{ijkdv}^{E_{c}}-\eta s_{ijkdv}^{c}{\left[s_{ijkdu}^{c}\right]}^{+}\right)\text{​}.$$

In (19), *u* and *v* are the two branches of orientation *k*, δ_*I*_ is a large gain that ensures rapid response of the inhibitory interneuron, and η = 1. Term *H*^*E*_*c*_^_*ijkdv*_ from the long-range connection excites the interneuron, whereas term $-\eta s_{ijkdv}^{c}{\left[s_{ijkdu}^{c}\right]}^{+}$ defines the recurrent inhibition that normalizes the total inhibitory activity, thereby enabling the 2-to-1 bipole interaction. Solving (19) at equilibrium yields the steady-state inhibitory activities that are used in the simulations:

(20) $$s_{ijkdv}^{c}=\left(-B_{v}+\sqrt{B_{v}^{2}+4\eta H_{ijkdv}^{Ec}}\right)/2\eta ,$$

and

(21) $$s_{ijkdu}^{c}=\left(-B_{u}+\sqrt{B_{u}^{2}+4\eta H_{ijkdu}^{Ec}}\right)/2\eta .$$

In (20) and (21), respectively, $B_{v}=1+\eta \left(H_{ijkdu}^{Ec}-H_{ijkdv}^{Ec}\right)$ and $B_{u}=1+\eta \left(H_{ijkdv}^{Ec}-H_{ijkdu}^{Ec}\right)$. A bipole cell will not fire when it receives excitatory input from only one side of its long-range connection, but it can fire when it receives excitatory inputs from both sides. In (19), for example, when *H*^*Ec*^_*ijkdu*_ equals zero, then *s*^*c*^_*ijkdu*_ equals zero. As a result, *s*^*c*^_*ijkdv*_ equals *H*^*Ec*^_*ijkdv*_. The total excitatory input from the long-range connections then equals the total inhibitory input from the inhibitory interneurons, and cancel. However, when the collinear excitatory long-range recurrent inputs *H*^*Ec*^_*ijkdu*_ and *H*^*Ec*^_*ijkdv*_ from both sides of the target cell are far from zero, the sum of these excitatory inputs is larger than the total self-normalizing inhibitory input, and thus the cell can fire if the cell if it is in V2 see Equation (30). If the cell is in V1, as in Equation (13), then the excitatory inputs from its long-range connections are modulatory, thereby enhancing bipole cell firing only if it also receives excitatory input from layer 4.

Term γ_3_[*c*_*ijkd*_ − β_*c*_]^+^ in (13) is self-excitatory feedback, with threshold β_*c*_ = 0.03. Term *C*^*P*^_*ijkd*_ is the inhibitory input at the same position and disparity from other bipole cells that code different orientations:

(22) $$C_{ijkd}^{P}=\gamma_{4}\left(\sum_{r\neq k}{\left[c_{ijrd}-\beta_{c}\right]}^{+}\right),$$

where γ_4_ = 5. Term *C*^*S*^_*ijkd*_ is the inhibitory input from spatial competition across position and orientation, but within disparity. Competition across position sharpens and localizes the spatial positions of boundaries. Competition across orientation prevents abutting lines in the image of different orientations from generating illusory contours that can penetrate the interior of a differently oriented boundary. This latter property is called *spatial impenetrability* (Grossberg and Mingolla, 1987; Grossberg and Williamson, 2001). Term *C*^*S*^_*ijkd*_ obeys:

(23) $$C_{ijkd}^{S}=\gamma_{5}\sum_{p\neq i,q\neq j,r}W_{pqijk}{\left[c_{pqrd}-\beta_{c}\right]}^{+}.$$

In (23), γ_5_ = 1, and *W*_*pqijk*_ is an elliptic Gaussian kernel elongated at the orientation perpendicular to *k*. For the *W*_*pqijk*_ vertical orientation (*k* = 1):

(24) $$W_{pqij1}=\exp\text{​}\left(-\left(\frac{{\left(i-p\right)}^{2}}{\sigma_{p}^{2}}+\frac{{\left(j-q\right)}^{2}}{\sigma_{q}^{2}}\right)\right),$$

where σ_*p*_ > σ_*q*_ (σ_*p*_ = 8, σ_*q*_ = 0.3). The horizontally oriented kernel is obtained by rotation.

The membrane potential, *c*^*L/R*^_*ijk*_, of a monocular complex cell obeys the same equation as that of a binocular complex cell *c*_*ijkd*_:

(25) $$\begin{array}{l} \frac{d}{dt}c_{ijk}^{L/R}=-\alpha c_{ijk}^{L/R}+\left(\beta -c_{ijk}^{L/R}\right)\\ \;\left(I_{ijk}^{c,L/R}\left(\gamma_{1}+\gamma_{2}{\left[\sum_{v}H_{ijkv}^{E_{c}}-H_{ijk}^{I_{c}}\right]}^{+}\right)+\gamma_{3}{\left[c_{ijk}^{L/R}-\beta_{c}\right]}^{+}\right)\\ \;-\left(1+c_{ijk}^{L/R}\right)\left(C_{ijk}^{P}+C_{ijk}^{S}\right). \end{array}$$

The input *I*^*c,L/R*^_*ijk*_ from V1 layer 3B monocular simple cells obeys:

(26) $$I_{ijk}^{c,L/R}={\left[b_{ijk}^{L/R,+}-\theta \right]}^{+}+{\left[b_{ijk}^{L/R,-}-\theta \right]}^{+}.$$

The parameters α = 20,β = 8, and θ = 0.4. The other parameters are the same as the binocular parameters.

### 4.6. V2 layer 4

Most V2 cells are binocular (Hubel and Livingstone, 1987), consistent with the model's combination of V1 left and right monocular inputs with binocular inputs in layer 4 of V2, such that monocular inputs are added to all depth planes along their respective lines–of–sight, yielding. Initially, the following initial membrane potential, *v*_*ijkd*_, of a V2 layer 4 cell:

(27) $$\begin{array}{l} v_{ijkd}=\alpha h\left({\left[c_{ijkd}^{-}\theta \right]}^{+}\right)+\beta (h\left({\left[c_{(i+s)jk}^{L}-\theta_{m}\right]}^{+}\right)\\ \;+h\left({\left[c_{(i-s)jk}^{R}-\theta_{m}\right]}^{+}\right)). \end{array}$$

In (27), *s* is the positional shift between left and right eye input (see Table 1); *h* is a signal function with *h(x)* = 1 if *x* > 0, 0 otherwise; Θ and θ_*m*_ are signal thresholds (0.06, 0.3); and *α* and β (2.6, 0.8) are the gains of the excitatory binocular and monocular connections, respectively.

These layer 4 boundary cells in the V2 pale stripes also receive feedback signals from left and right monocular surfaces in the V2 thin stripes. These are the surface-to-boundary surface contour feedback signals that were discussed in constraint (7) of Section 1. When these surface contour signals are active, (27) is replaced, at steady-state, by:

(28) $$\begin{array}{l} v_{ijkd}=(\alpha h\left({\left[c_{ijkd}-\theta \right]}^{+}\right)+\beta (h\left({\left[c_{(i+s)jk}^{L}-\theta_{m}\right]}^{+}\right)\\ \;+h\left({\left[c_{(i-s)jk}^{R}-\theta_{m}\right]}^{+}\right)))(1+\alpha_{f}f_{ijkd})\\ \;\left(\delta +(1-\delta )h(f_{ijkd})\right). \end{array}$$

In (28), *f*_*ijkd*_ is the total surface contour feedback signal, α_*f*_ is its excitatory gain (1.1), and α, β, *h*, θ and θ_*m*_ are the same as in (27). Parameter δ (0.2) scales the activities of layer 4 cells. If δ < 1, then the activities of layer 4 cells that do not receive surface contours signals are suppressed to some degree. The total surface contour feedback signal *f*_*ijkd*_ obeys:

(29) $$f_{ijkd}={\left[f_{ijkd}^{L}-\theta_{f}\right]}^{+}+{\left[f_{ijkd}^{R}-\theta_{f}\right]}^{+}.$$

In (29), *f*^*L*^_*ijkd*_ and *f*^*R*^_*ijkd*_ are the surface contour signals derived from left and right monocular surfaces, respectively, and θ_*f*_ is a threshold (0.03).

### 4.7. V2 layer 2/3 complex cells

The bipole cells in V2 layer 2/3 implement perceptual grouping by long-range horizontal connections, and include the disparity filter as part of the inhibitory interactions that control perceptual grouping. See constraint (5) of Section 1. The membrane potential, *g*_*ijkd*_, of the bipole cell at position (i,j) that codes orientation k and disparity d obeys:

(30) $$\begin{array}{l} \frac{d}{dt}g_{ijkd}=-\alpha g_{ijkd}+(\beta -g_{ijkd})\\ \;\left(\gamma_{1}I_{ijkd}^{g}+\gamma_{2}{\left[\sum_{v}H_{ijkdv}^{E_{g}}-H_{ijkd}^{I_{g}}\right]}^{+}\right)\\ \;-(1+g_{ijkd})G_{ijkd}^{P}, \end{array}$$

where α, β, γ_1_, and γ_2_ are constants (α = 30, β = 10, γ_1_ = 1.4, γ_2_ = 1). Term *I*^*g*^_*ijkd*_ in (30) is the input from V2 layer 4:

(31) $$I_{ijkd}^{g}={\left[v_{ijkd}\right]}^{+}.$$

As for V1 layer 2/3 cells, V2 layer 2/3 bipole cells receive long-range recurrent excitatory signals from other (almost) collinear and coaxial bipole cells at nearby positions with the same disparity preference. Term *H*^*E*_*g*_^_*ijkdv*_ is the total excitatory input from branch *v* of the bipole cell at position (*i,j*), orientation *k* and disparity *d*:

(32) $$H_{ijkdv}^{Eg}=\sum_{pq}W_{pqijkv}^{g}{\left[g_{pqkd}-\zeta_{g}\right]}^{+}.$$

The long-range connection weight *W*^*g*^_*pqijkv*_ is the same as *W*^*c*^_*pqijkv*_ in (16) and (17), but with a larger spatial range (diameter = 7) and σ_*p*_ = 15, σ_*q*_ = 0.1, and ζ_*g*_ = 0.03. Term *H*^*Ig*^_*ijkd*_ is the total input from the inhibitory interneurons:

(33) $$H_{ijkd}^{Ig}=\sum_{v}{\left[s_{ijkdv}^{g}\right]}^{+}.$$

The activity, *s*^*g*^_*ijkdv*_, of the inhibitory interneuron for branch *v* has the same form as the inhibitory interneurons that are defined in Equation (19) for V1 layer 2/3:

(34) $$\frac{d}{dt}s_{ijkdv}^{g}=\delta_{I}\left(-s_{ijkdv}^{g}+H_{ijkdv}^{E_{g}}-\eta s_{ijkdv}^{g}{\left[s_{ijkdu}^{g}\right]}^{+}\right).$$

The parameters in (34) are also the same as those in (19).

Term *G*^*P*^_*ijkd*_ in (30) is the disparity filter (DF in Figure 1A) whose inhibitory signals cross disparities along the lines-of-sight:

(35) $$\begin{array}{l} G_{ijkd}^{P}=\gamma_{3}\sum_{d^{\prime }\neq d}M_{dd^{\prime }}({\left[g_{(i+s^{\prime }-s)jkd^{\prime }}-\beta_{g}\right]}^{+}\\ \;+{\left[g_{(i+s-s^{\prime })jkd^{\prime }}-\beta_{g}\right]}^{+}). \end{array}$$

In (35), γ_3_ is the gain (5) of the total disparity filter inhibition; *M*_*dd*′_ is the connection strength of line-of-sight inhibition from all cells that share a monocular input between disparities *d* and *d′* (see Table 2); and [*g*_(*i* + *s*′ − *s*)*jkd*′_ − β_*g*_]^+^ and [*g*_(*i* + *s* − *s*′)*jkd*′_ − β_*g*_]^+^ are V2 layer 2/3 bipole cell inhibitory inputs along the left and right lines-of-sight with the corresponding disparity-induced positional shifts *s* and *s′* (see Table 1) and threshold β_*g*_ (0.03).

**Table 2:** **The inhibition coefficients *M*_*dd*′_ that define line-of-sight inhibition**.

	**V. Near**	**Near**	**Zero**	**Far**	**V. Far**
V. Near	–	3	5	3	2
Near	0.4	–	2.5	2	0.4
Zero	0.3	1.5	–	1.5	0.3
Far	0.4	2	2.5	–	0.4
V. Far	2	3	5	3	–

*Each neuron is inhibited by every other neuron that shares either of its inputs by an amount that depends on the disparities of the inhibited and inhibiting neurons (cf. Figure 3)*.

The disparity filter (*G*^*P*^) works across a range of parameter values. As illustrated in Table 2, it needs to be symmetrical about the fixation plane (i.e., the near and far disparity planes equally inhibit and are equally inhibited by the zero disparity plane), and the zero disparity plane inhibits the near and far disparity planes more than conversely.

### 4.8. V2 thin stripe monocular surface filling-in

Monocular surface filling-in occurs within V2 thin stripes in response to lightness signals from LGN via V1 blobs, and binocular boundary signals from V2 layer 2/3 bipole cells. The boundary signals create resistive barriers that contain the spread of lightness during the filling-in process. The activity *F*^*L/R*^_*ijd*_ in each Filling-In Domain, or FIDO, is the membrane potential of a left (L) or right (R) monocular surface cell at position (*i,j*) and disparity *d*. These activities obey a nearest-neighbor diffusion equation (Grossberg and Todorović, 1988):

(36) $$\varepsilon \frac{d}{dt}F_{ijd}^{L/R}=-\alpha F_{ijd}^{L/R}+\sum_{(p,q)\in N_{ij}}\left(F_{pqd}^{L/R}-F_{ijd}^{L/R}\right)\Phi_{pqijd}+x_{ijd}^{L/R}.$$

The rate parameter ε << 1 ensures that this surface filling-in process within the thin stripes is faster than the boundary grouping process within the pale stripes. Parameter α is the decay rate (1.0); and *N*_*ij*_ is the set of the nearest-neighbor positions of (*i,j*):

(37) $$N_{ij}=\left\{\left(i,j-1\right),\left(i-1,j\right),\left(i+1,j\right),\left(i,j+1\right)\right\};$$

and *x*^*L/R*^_*ijd*_ is the lightness input from the left (L) or right (R) LGN:

(38) $$x_{ijd}^{L}={\left[x_{(i+s)j}^{L}\right]}^{+}$$

and

(39) $$x_{ijd}^{R}={\left[x_{(i-s)j}^{R}\right]}^{+},$$

where *s* is the disparity-sensitive positional shift (see Table 1). The boundary-gated diffusion coefficients, Φ_*pqijd*_, in (36) are suppressed at positions where the boundary signals are large:

(40) $$\begin{array}{l} \Phi_{pqijd}=\frac{\delta }{1+\rho \left(g_{(i-0.5)(j-0.5)d}+g_{(i-0.5)(j+0.5)d}\right)}\\ \text{ if p}=i-1\text{ and q}=j, \end{array}$$

(41) $$\begin{array}{l} \Phi_{pqijd}=\frac{\delta }{1+\rho \left(g_{(i+0.5)(j-0.5)d}+g_{(i+0.5)(j+0.5)d}\right)},\\ \text{ if p}=i+1\text{ and q}=j, \end{array}$$

(42) $$\begin{array}{l} \Phi_{pqijd}=\frac{\delta }{1+\rho \left(g_{(i-0.5)(j-0.5)d}+g_{(i+0.5)(j-0.5)d}\right)},\\ \text{ if p}=i\text{ and q}=j-1, \end{array}$$

(43) $$\begin{array}{l} \Phi_{pqijd}=\frac{\delta }{1+\rho \left(g_{(i-0.5)(j+0.5)d}+g_{(i+0.5)(j+0.5)d}\right)},\\ \text{ if p}=i\text{ and q}=j+1. \end{array}$$

The diffusion parameter δ = 2000, and the boundary-gating parameter ρ = 200. The boundary terms in these equations sum over all orientations of bipole cell activations at the corresponding position and disparity:

(44) $$g_{ijd}=\gamma \sum_{k}{\left[g_{ijkd}-\theta_{g}\right]}^{+},$$

with gain γ = 10 and threshold θ_*g*_ = 0.03.

In Equations (40–(43), the lattice of boundary-gating signals is offset by [0.5, 0.5] relative to the lattice of discounted lightness inputs. This enables the boundary-gating signals to contain filling-in without trapping the lightness inputs within the boundary itself. These two processing streams are also spatially displaced in the cortical map. Spurious edge effects were avoided by using wrap–around whereby the last element of a row/column is adjacent to the first element of the same row/column (Grossberg and Howe, 2003).

The steady-state of (36) is used in the simulations; namely:

(45) $$F_{ijd}^{L/R}=\frac{x_{ijd}^{L/R}+\sum_{(p,q)\in N_{ij}}F_{pqd}^{L/R}\Phi_{pqijd}}{\alpha +\sum_{(p,q)\in N_{ij}}\Phi_{pqijd}}.$$

This approximation is justified by the assumption of filling-in that is fast relative to the rate of boundary formation. During each time step of boundary grouping, monocular surfaces are filled-in using (45) and generate surface-to-boundary surface contour signals before the process is reiterated.

### 4.9. Surface contour feedback signals

The surface contour signals project from V2 monocular surfaces in the thin stripes to V2 layer 4 cells in the pale stripes to modulate binocular boundaries in layer 2/3; see Equation (28). Output signals from the left (L) and right (R) monocular surface activities (45) are derived from oriented filters:

(46) $$f_{ijkd}^{L/R,+}=\sum_{p,q}{\text{K}}_{pqk}^{}{\left[F_{i+p,j+q,d}^{L/R}\right]}^{+},$$

(47) $$f_{ijkd}^{L/R,-}=-f_{ijkd}^{L/R,+}=-\sum_{p,q}{\text{K}}_{pqk}{\left[F_{i+p,j+q,d}^{L/R}\right]}^{+}.$$

where the Gabor kernel K_*pqk*_ is defined in Equation (7). Their sum defines the final surface contour signals:

(48) $$f_{ijkd}^{L/R}={\left[f_{ijkd}^{L/R,+}\right]}^{+}+{\left[f_{ijkd}^{L/R,-}\right]}^{+}.$$

V4 binocular surface filling-in: Visible 3D percepts. V4 is predicted in the model to support visible percepts of 3D surfaces. To accomplish this, V4 receives lightness signals from the LGN via V1 blobs and V2 thin stripes, and boundary signals from V2 layer 2/3. It combines the monocular lightness signals from the two eyes that correspond to the same 3D location. Its binocular lightness input, *z*_*ijd*_, sums the rectified monocular lightness signals from the left (L) and right (R) eyes that correspond to the same 3D position:

(49) $$z_{ijd}={\left[x_{(i+s)j}^{L}\right]}^{+}+{\left[x_{(i-s)j}^{R}\right]}^{+}.$$

In (49), *i, j* are positional indices, *d* disparity and *s* the positional shift defined in Table 1. V4 cell membrane potentials, *w*_*ijd*_, undergo binocular surface filling-in using a steady-state diffusion equation similar to (36):

(50) $$w_{ijd}=\frac{z_{ijd}+\sum_{(p,q)\in N_{ij}}w_{pqd}\Phi_{pqijd}}{\alpha +\sum_{(p,q)\in N_{ij}}\Phi_{pqijd}}.$$

In (50), parameter *α* = 1; the set of nearest-neighbors *N*_*ij*_ is defined in (A37); and Φ_*pqijd*_ is defined in (40)–(43) with the diffusion parameter δ = 1000 and the boundary-gating parameter ρ = 400.

### 4.10. Obligate theorem

The following theorem shows that the obligate property holds at the binocular simple cells in layer 3B. See Grossberg and Howe (2003) for a proof.

**Obligate Theorem**. Consider the system:

(51) $$\begin{array}{l} \frac{db_{ijkd}^{B,+}}{dt}=-\gamma_{1}b_{ijkd}^{B,+}+\left(1-b_{ijkd}^{B,+}\right)\left(S_{(i+s)jk}^{L,+}+S_{(i-s)jk}^{R,+}\right)\\ \;-\alpha \text{​}\left({\left[q_{ijkd}^{L,+}\right]}^{+}\text{​}+{\left[q_{ijkd}^{L,-}\right]}^{+}\text{​}+{\left[q_{ijkd}^{R,+}\right]}^{+}\text{​}+{\left[q_{ijkd}^{R,-}\right]}^{+}\right), \end{array}$$

(52) $$\begin{array}{l} \frac{dq_{ijkd}^{L,+}}{dt}=-\gamma_{2}q_{ijkd}^{L,+}+S_{(i+s)jk}^{L,+}\\ \;-\beta \left({\left[q_{ijkd}^{R,+}\right]}^{+}+{\left[q_{ijkd}^{R,-}\right]}^{+}+{\left[q_{ijkd}^{L,-}\right]}^{+}\right)\text{​}, \end{array}$$

(53) $$\begin{array}{l} \frac{dq_{ijkd}^{R,+}}{dt}=-\gamma_{2}q_{ijkd}^{R,+}+S_{(i-s)jk}^{R,+}\\ \;-\beta \left({\left[q_{ijkd}^{L,+}\right]}^{+}+{\left[q_{ijkd}^{L,-}\right]}^{+}+{\left[q_{ijkd}^{R,-}\right]}^{+}\right)\text{​}, \end{array}$$

(54) $$\begin{array}{l} \frac{dq_{ijkd}^{L,-}}{dt}=-\gamma_{2}q_{ijkd}^{L,-}+S_{(i+s)jk}^{L,-}\\ \;-\beta \left({\left[q_{ijkd}^{R,-}\right]}^{+}+{\left[q_{ijkd}^{R,+}\right]}^{+}+{\left[q_{ijkd}^{L,+}\right]}^{+}\right)\text{​}, \end{array}$$

and

(55) $$\begin{array}{l} \frac{dq_{ijkd}^{R,-}}{dt}=-\gamma_{2}q_{ijkd}^{R,-}+S_{(i-s)jk}^{R,-}\\ \;-\beta \left({\left[q_{ijkd}^{L,-}\right]}^{+}+{\left[q_{ijkd}^{L,+}\right]}^{+}+{\left[q_{ijkd}^{R,+}\right]}^{+}\right), \end{array}$$

where

(56) $$S_{(i+s)jk}^{L,+/-}={\left[s_{(i+s)jk}^{L,+/-}-\theta \right]}^{+},$$

and

(57) $$S_{(i-s)jk}^{R,+/-}={\left[s_{(i-s)jk}^{R,+/-}-\theta \right]}^{+}.$$

In (6) and (7), *s*^*L*,+/−^_(*i* + *s*)*jk*_ and *s*^*R*,+/−^_(*i* − *s*)*jk*_ are the monocular simple cell activities defined by (6) and (8). The parameters θ ≥ 0, γ_1_ > 0 and

(58) $$0<\beta <\gamma_{2}<\alpha <\gamma_{2}+\beta$$

Then, in response to constant inputs, the system converges exponentially to the following unique equilibrium. Let Γ = γ_1_ + *S*^*L*,+^_(*i* + *s*)*jk*_ + *S*^*R*,+^_(*i* − *s*)*jk*_.

$$(1)\text{ if }0<S_{(i+s)jk}^{L,+},S_{(i-s)jk}^{R,+};\text{ and }\frac{\beta }{\gamma_{2}}\leq \frac{S_{(i+s)jk}^{L,+}}{S_{(i-s)jk}^{R,+}}\leq \frac{\gamma_{2}}{\beta },$$

then at equilibrium

(59) $$b_{ijkd}^{B,+}=\frac{1}{\Gamma }\left(1-\frac{\alpha }{\gamma_{2}+\beta }\right)\left(S_{(i+s)jk}^{L,+}+S_{(i-s)jk}^{R,+}\right);$$

$$(2)\text{ if }0<S_{(i+s)j}^{V,L,+},S_{(i-s)j}^{V,R,+};\text{ and }\frac{S_{(i+s)jk}^{L,+}}{S_{(i-s)jk}^{R,+}}>\frac{\gamma_{2}}{\beta_{2}},$$

then at equilibrium

(60) $$b_{ijkd}^{B,+}=\frac{1}{\Gamma }\left(S_{(i-s)jk}^{R,+}+\left(1-\frac{\alpha }{\gamma_{2}}\right)S_{(i+s)jk}^{L,+}\right);$$

$$(3)\text{ if }0<S_{(i+s)j}^{V,L,+},S_{(i-s)j}^{V,R,+};\text{ and }\frac{S_{(i+s)jk}^{L,+}}{S_{(i-s)jk}^{R,+}}<\frac{\beta }{\gamma_{2}},$$

then at equilibrium

(61) $$b_{ijkd}^{B,+}=\frac{1}{\Gamma }\left(S_{(i+s)jk}^{L,+}+\left(1-\frac{\alpha }{\gamma_{2}}\right)S_{(i-s)jk}^{R,+}\right);$$

(62) $$\begin{array}{l} (4)\text{ for all other values of }S_{(i+s)jk}^{L,+},S_{(i-s)jk}^{R,+},\\ \text{at equilibrium }b_{ijkd}^{B,+}\leq 0. \end{array}$$

## 5. Discussion: conscious 3D surface percepts are part of surface-shroud resonances

The current simulations illustrate how the interactions among identified neurons in laminar cortical circuits can give rise to consciously seen surface percepts such as the Venetian blind and Panum's limiting case percepts. These simulations, along with those in articles such as Cao and Grossberg (2005, 2012); Fang and Grossberg (2009), Grossberg and Howe (2003), Grossberg et al. (2007), Grossberg and McLoughlin (1997), Grossberg and Raizada (2000), Grossberg and Swaminathan (2004), Grossberg and Yazdanbakhsh (2005), Kelly and Grossberg, 2000, and Raizada and Grossberg (2001), show how the model cortical interactions of FACADE theory and its laminar cortical extension in the 3D LAMINART model provide unified explanations, and testable predictions, about a wide variety of psychophysical and neurobiological data about 2D and 3D perceptual grouping, surface perception, and figure-ground separation.

The filled-in surface representations in all of these articles have parametric properties that closely match visually perceived and reported surface percepts by human subjects. It is for this reason that the liberty is taken of calling them model representations of conscious percepts. However, the model as stated in the current article is insufficient to represent the dynamics that may subserve a conscious percept in the brain. This insufficiency may be better understood from the vantage point of other theoretical results which clarify how the current model may be consistently embedded into a larger theory wherein more sophisticated correlates of conscious events may be represented.

This insufficiency may be summarized in the light of two theoretical predictions. The first prediction is part of FACADE theory (Grossberg, 1994) and the 3D LAMINART model. It claims that “conscious visual percepts are surface percepts” that are represented within the surface cortical stream through V1 blobs, V2 thin stripes, and their projections to V4 and beyond. This prediction coexists with the companion prediction that “all boundaries are invisible” within the boundary cortical stream through V1 interblobs, V2 pale stripes, and their projections to V4 and beyond.

The second prediction is part of Adaptive Resonance Theory, or ART (Grossberg, 1976, 2012, 2013; Carpenter and Grossberg, 1991, 1993). It claims that “all conscious states are resonant states.”

Putting together these two predictions raises the question: what resonance enables filled-in surface representations to be consciously seen? Recent research on how the brain coordinates attention, perception, eye movement search, learning, and recognition of invariant object categories, embodied in a class of models whose variations are called ARTSCAN, distributed ARTSCAN (dARTSCAN), positional ARTSCAN (pARTSCAN), and ARTSCAN Search (Fazl et al., 2009; Grossberg, 2009; Cao et al., 2011; Foley et al., 2012; Chang et al., 2014), has shed new light on this question. In particular, these models have explained and predicted how surface representations, say in cortical area V4, bid for spatial attention, say in posterior parietal cortex (PPC), and how a winning surface, to which focused spatial attention is paid, enables spatial attention in PPC to fit itself to the shape of the surface. Such form-fitting spatial attention is sometimes called an *attentional shroud* (Tyler and Kontsevich, 1995) and the feedback interaction that maintains spatial attention in PPC upon the surface of interest in V4 is said to form a *surface-shroud resonance*.

Grossberg (2012, 2013) has predicted that every conscious visual surface percept is part of a surface-shroud resonance. Such a resonance has been proposed to propagate both top-down to lower cortical levels, such as V1, where finer features of seen representations may be represented, as well as bottom-up to higher cortical areas. For purposes of the present article, it suffices to observe that the current 3D LAMINART model can be consistently embedded into a larger system for focusing spatial attention from the Where cortical stream upon consciously seen visual surface representations using surface-shroud resonances, while learning to recognize them in the What cortical stream using feature-category resonances, such as those modeled by ART (Grossberg, 1980, 2012; Carpenter and Grossberg, 1991, 1993). Said more simply, a mechanistic account is now available for documenting the differences between seeing and knowing, and how they are coordinated into seamless moments of conscious awareness using interactions between the What and Where cortical streams.

### Conflict of interest statement

The authors declare that the research was conducted in the absence of any commercial or financial relationships that could be construed as a potential conflict of interest.
